# Bis-pyrrolidone structures as versatile building blocks for the synthesis of bio-based polyesters and for the preparation of additives†

**DOI:** 10.1039/d4gc04951a

**Published:** 2025-01-03

**Authors:** Nele Schulte, Giacomo Damonte, Valeria Marisa Rocca, Anamaria Todea, Orietta Monticelli, Alessandro Pellis

**Affiliations:** a Università degli Studi di Genova, Dipartimento di Chimica e Chimica Industriale via Dodecaneso 31 16146 Genova Italy alessandro.pellis@unige.it; b Faculty of Industrial Chemistry and Environmental Engineering, Polytechnic University of Timişoara Carol Telbisz 6 300001 Timişoara Romania

## Abstract

In this work, three bis-pyrrolidone-based structures (BP) were synthesized combining dimethyl itaconate (DMI), the dimethyl ester derivative of itaconic acid, with various aliphatic diamines having a C4 to C12 carbon chain length with the aim of developing novel bio-based building blocks. All three BPs were obtained with a purity >93% and could further be used without performing any tedious purification step, therefore allowing an easy scalability of the synthesis on a 10 g scale. Their potential application was demonstrated in two key areas of modern polymer science: (1) the enzymatic synthesis of polyesters and (2) their use as poly(lactic acid) (PLA) additives. Firstly, the possibility of obtaining oligoesters by reacting the BP monomers with various aliphatic diols in a solventless reaction system and under mild conditions (*T* < 90 °C) was demonstrated thanks to the use of enzymatic catalysis. Linear oligoesters having mean average molecular weights between 1000 g mol^−1^ and 6100 g mol^−1^ and dispersity values <2 were successfully obtained. When applying the BP structures as PLA additives, the incorporation of a 10% w w^−1^ BP in the polyester matrix resulted in systems with an 8× increased elongation at break and a decrease in the glass transition temperature compared to the neat polymer matrix.

## Abstract 2

Facile enzymatic synthesis of bis-pyrrolidone structures from dimethyl itaconate and various aliphatic diamines (C4–C12) and their use for the enzymatic synthesis of oligoesters and the plasticization of poly(lactic acid).

## Graphical abstract

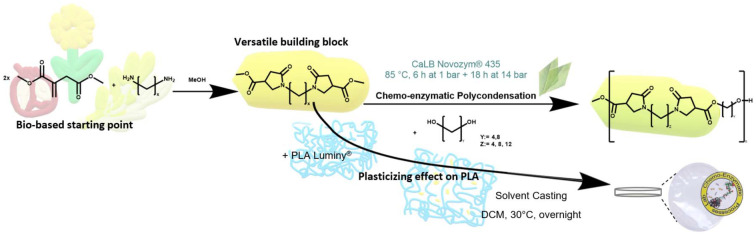

**Green foundation**

1. Showing the possibility of combining traditional chemistry with biocatalysis in a novel interdisciplinary approach that is applied to polymer chemistry

2. The development of a chemo-enzymatic strategy for the synthesis of bio-based additives

3. Varying the structures of the diamines used in this work to prepare novel architectures having different properties

## Introduction

Itaconic acid (IA) has been receiving attention already since the 1950s because of its use as a building block for bio-based polymers and additives.^1,2^ IA is mainly produced *via* fermentation of the lignocellulosic feedstock as the synthetic route is not efficient enough compared to the fermentative approach. Since a significant amount of IA can be obtained from various sugars and alcohols, the platform chemical was rated as one of the 12 most promising building blocks by the US Department of Energy.^3^ The market of IA is expected to grow from USD 101.4 million in 2022 to USD 110.4 million by 2028 with a compound annual growth rate (CAGR) of 7.8%.^4–6^ The derivatization of IA and its use as a platform chemical have been extensively described in a variety of fields, including antimicrobial agents, cosmetics, water treatment, adhesives, synthetic resins, medicines, insecticides, and optical materials.^7–10^ This important raw material is used either in the form of itaconic-based salts or esters. As an interesting example, Dai *et al.* published a case study for coating applications using IA as the crosslinker in a radical polymerization reaction.^11^ Indeed, due to the structural resemblance of IAs with acrylic and methacrylic acids, it has great potential as a renewable substitute in radiation-curing binders for coating and printing ink applications. In addition, over the past 30 years, IA has been used for polymer synthesis *via* traditional metal- and acid-based catalysis.^10–13^ As one of the first research groups to attempt polymer synthesis using IA, Singh *et al.* synthesized polyesters from IA and PEG-600.^10^ The reaction was performed at 150 °C for 22 h under vacuum by applying *p*-toluenesulfonic acid as the catalyst and hydroquinone as the inhibitor to prevent radical crosslinking of the unsaturated double bonds.^10^ The resulting polyesters were then used as building blocks for biodegradable hydrogel microspheres loaded with vaccines. Recently, an increasing number of publications have additionally described polycondensation *via* enzyme catalysis and functionalization.^12,14,15^ Pellis *et al.* reported the synthesis of polyesters with a molecular weight of 2600 g mol^−1^ when combining DMI with 1,8-octanediol (ODO).^14^ Similarly, Barrett *et al.*, who focused on the synthesis of biomaterials for biomedical and biotechnological applications, were able to produce linear polyesters from various diols with molecular weights ranging from 200 to 11 900 g mol^−1^, while Jiang *et al.* reported extremely high molecular weights of up to 58 000 g mol^−1^, both using Lipase B from *Candida antarctica* (CaLB).^12,14,15^ Not surprisingly, the IA to adipic acid ratio significantly affected the characteristics of the prepared materials with the reactions that were run for 94 h at a relatively low reaction temperature (90 °C).^12^ The polyesters prepared by Jiang *et al.* were co-polyesters from 1,4-butanediol, IA, and a second saturated dicarboxylic acid under similar synthetic conditions to those employed by Barrett *et al.* An enzymatic polymerization was then carried out using a two-stage procedure.^12,15^ The first step took effect for two hours at 80 °C in a nitrogen environment. Subsequently, the second step was conducted for an additional 94 h at 80 °C with a lower pressure.^15^ The reaction conditions applied by Pellis *et al.* were shorter (24 h) and focused on avoiding undesired side reactions.^14^

**Fig. 1:**
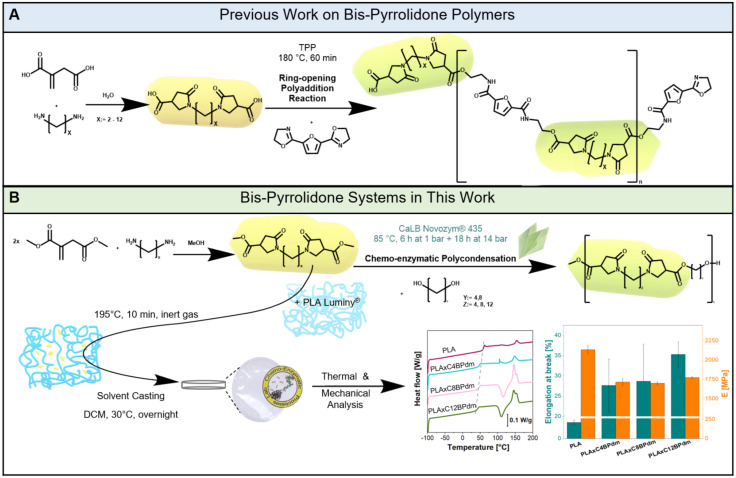
Scheme of the synthesis of bis-pyrrolidone (BP)-based polymers. (A) The BP-based polyaddition reaction previously reported by Roy *et al.* (mechanism adapted from Roy *et al.*, 2018)^29^ and (B) the enzymatically synthesized C*x*BPdm-based polyester structure and poly(lactic acid) plasticization experiments carried out in this work. Representative graphs for mechanical and thermal analysis are given (see also Fig. 5, and Fig. S32 in the ESI†).

Novozym 435 CaLB is a widely recognized lipase immobilized biocatalyst.^16,17^ Thanks to its robust catalytic activity in ester bond formation through transesterification and esterification, it has become one of the most attractive lipases for catalysis in polymer chemistry. The polycondensation reaction is driven by its active site containing a serine–histidine–aspartate catalytic triad (Ser105, His224 and Asp187).^17–19^ The hydrophobic surface around the active site aids the diffusion of CaLB's substrates into the enzyme's binding site pocket. A separation of functionality is introduced to the pocket through the two small channels on either side. One channel functions as the acyl acceptor, harboring the corresponding acrylic side of the substrate, whereas the other contains the alcohol.^20^ The catalytic triad facilitates the nucleophilic attack by activating hydroxyl or amino groups from the diols or diamines on the electrophilic carbonyl group of the ester substrate. The nucleophilic attack on the carbonyl carbon of the ester group leads to the formation of an acyl-enzyme complex where the ester group is covalently bound to Ser105. The diol's hydroxyl group acts as the second nucleophile. The formation of the new ester bond is possible as the acyl-enzyme complex is attacked. As the enzyme can work under solvent-free and mild conditions (low temperature and pressure) when employing CaLB as the catalyst it makes the process highly sustainable and ideal for green polymer synthesis.^14,21^ Furthermore, the mild conditions can minimize side reactions, reduce energy consumption, and eliminate the need for radical inhibitors or other additives commonly required in traditional polymerization methods.^22,23^ Additionally, the enzymatic approach circumvents the use of toxic catalysts, aligning with green chemistry principles and enhancing the sustainability of the process.^24^ Undesirable crosslinked materials can be avoided in chemoenzymatic polymerization by CaLB as shown recently for glycerol-based oligomers while maintaining high specificity and efficiency.^25^ Similarly, enzymatic polymerization of IA and its derivatives has shown excellent control over polymer architecture, further underlining the value of biocatalysis in the development of advanced, environmentally friendly polymers.^14^ An example of a newly established branch of the use of IA is the synthesis of bis-pyrrolidone-type monomers. Indeed, the properties of bis(pyrrolidone)-based structures have shown promise for application as compatibilizers and additives.^26,27^ Obtained from the reaction between IA or its diester with aliphatic spacer units (such as diamines of various lengths), the moieties can be used in polycondensation with diamines or diols and are therefore of great interest in the context of the development of biopolymer-based formulations.^26–29^ In the case of Dai *et al.*'s work, the synthesis was carried out using three distinct primary diols as monomers and two different catalysts.^11^ In the presence of 0.5% w w^−1^*p*-toluenesulfonic acid, the first prepolymers were created. After 2 hours, 1% w w^−1^ of dibutyltin dilaurate was added as a second catalyst to promote transesterification, and water was drained under vacuum. Additionally, 0.5% w w^−1^ inhibitor, 4-methoxyphenol, was employed to prevent crosslinking of the unsaturated double bond of IA.^11^ An IA-based bis-pyrrolidone system for the synthesis of thermoset resins using a solely chemical approach has already been described (Fig. 1A). In 2024, Zhu and coworkers reported the successful synthesis of bis-pyrrolidone copolymers by melt polycondensation.^30^ Unlike the compared study, which achieved high molecular weight polymers (50 000–70 000 g mol^−1^) using antimony-based catalysts, the here presented work focuses on the enzymatic synthesis of short-chain oligomers. The use of CaLB as a catalyst eliminates the need for harmful metal-based catalysts and enabled the synthesis of short oligomers as bio-based additives was anticipated, rather than high molecular weight polymers, as described hereafter. These oligomers are particularly suited for applications as plasticizers, where their lower molecular weight facilitates integration into polymer matrices, enhancing flexibility and reducing the glass transition temperature (*T*_g_). The utilization of pyrrolidone-based building blocks presents several advantages over both traditional petroleum-based and other bio-based alternatives. The primary distinction lies in their bio-based origin, derived from renewable resources (dimethyl itaconate as a derivative of itaconic acid, which is mainly produced *via* fermentation).^22,31^ Moreover, itaconate-based polymer materials offer potential for biodegradability, contributing to reduced environmental impact at the end of the product lifecycle as shown in several recent studies.^30,32,33^ Compared to other bio-based building blocks, bis-pyrrolidone-systems stand out due to their synthetic route. The mild solvent-less process avoids harsh reaction conditions, toxic catalysts, and complex purification steps. This work focused on the preparation of various DMI-derived BP compounds (BPdm) with different lengths using three different aliphatic diamines. The synthesized compounds were applied as monomers to produce oligoesters by enzymatic catalysis and, for the first time, as additives for poly(lactic acid) (PLA) (Fig. 1B). Surprisingly, no study has investigated the use of BP compounds as biobased plasticizers for PLA, despite the fact that these compounds offer advantages in terms of ease of synthesis and sustainability. The development of PLA materials is facing limitations arising from the lack of compatibility between the respective constituents. Thus, investigation into novel bio-derivable plasticizers for PLA, such as the here used BP compounds, may be the start leading to further novel plasticization systems. The versatility of BPdm, which is shown by being applicable in the synthesis and modification of polymeric systems, is complemented by a facile synthesis. The novelty of this work lies in the solventless enzymatic catalysis employed to synthesize biobased oligomers from renewable precursors, demonstrating an eco-friendly and scalable approach to functional material development.

## Experimental section

### Materials

1,8-Octanediol (ODO, 98%), 1,4-butanediol (BDO, ReagentPlus®, 99%), 1,8-diaminooctane (DAO, 98%), ethyl acetate (EtOAc, for GC-MS, >99.5%), 2-methyltetrahydrofuran (MeTHF, stabilised with 2,6-di-*tert*-butyl-4-methylphenol, ≥98.0%), chloroform (for GPC, ≥99.5%) and dichloromethane (DCM, for solvent casting, ≥99.9%) were purchased from Merck. Dimethyl itaconate (DMI, 98%) and 1,12-diaminododecane (DADD, 98%) were purchased from TCI chemicals. Deuterated chloroform (for NMR, >99.8%, containing 0.03% (v/v) tetramethylsilane (TMS)) was purchased from Eurisotop. Acetone (99.8%) was purchased from VWR Chemicals (Vienna, Austria). Methanol (99.8%) and 1,4-diaminobutane (DAB, 99%) were purchased from Fluka Chemie GmbH (Buchs, Switzerland). Commercial poly(lactic acid) (PLA; Luminy® LX 175, 96%, *M*_w_ = 245 kg mol^−1^) was purchased from Corbion (Amsterdam, Netherlands). Novozym® 435 (product code: LC200 232) consisting of *Candida antarctica* lipase B immobilized on macroporous acrylic resin beads (CaLB) was obtained from Novozym® (Bagsvaerd, Denmark). All chemicals, enzymes, and reagents were used as received if not otherwise specified.

### Synthesis of bis-pyrrolidone moieties

The reactions were carried out on two scales. The small reaction set-up consisted of 5.0 mmol DMI, and 2.5 mmol aliphatic diamine (DAB, DAO, or DADD), and 0.3 mL (catalytic amount) methanol. The larger scale reaction was conducted with 25.0 mmol DMI and 12.5 mmol diamine (DAB, DAO, or DADD) and 1.5 mL methanol. Depending on the scale, the reactions were conducted in a 25 mL or a 50 mL round bottom flask, which was connected to a condenser to operate under reflux at 88 °C. The reaction mixtures were continuously stirred at 400 rpm using a magnetic flea on a Heidolph Hei-PLATE Mix ‘n’ Heat Core+ heating plate equipped with aluminium fitters (Heidolph, Germany). After 18 h, the reaction mixture was recovered without work-up. The excess methanol was then removed *via* rotary evaporation (R-3, BÜCHI) at 30 °C and subsequently with reduced pressure (4.5 mbar) on a high vacuum line equipped with a cold trap connected to an oil pump (Edwards RV3 oil filled rotary vane vacuum pump with FL20K front line trap/EMF10). The reaction products were then analyzed, without further purification steps, *via* Nuclear Magnetic Resonance (^1^H-NMR, ^13^C-NMR, and HSQC) spectroscopy, Fourier-transform infrared spectroscopy (FT-IR), thermo-gravimetric analysis (TGA), High Performance Liquid Chromatography (HPLC) coupled with Mass Spectroscopy, and Gas Chromatography Mass Spectrometry (GC-MS), as shown in Tables S1 and S2, Fig. S1 to S6, and S16 to S21 in the ESI.† The synthesized molecules are abbreviated as C*x*BPdm, where *x* indicates the type of diamine used based on the number of carbon atoms in the aliphatic chain while BPdm indicates that a bis-pyrrolidone structure is meant which carries methyl end groups on the two sides (*e.g.*: BPdm with 1,8-diaminooctane: C8BPdm).

### Enzymatic polycondensation reactions

Polycondensation was conducted as previously reported by our group.^34^ Briefly: reactions were performed in a solvent-less system using equimolar amounts (6.0 mmol) of carboxylic diester and diol using 10% w w^−1^ of biocatalyst, the commercial enzyme CaLB. The reactions were carried out at 85 °C on a Heidolph Hei-PLATE Mix‘n'Heat Core equipped with round bottom flask aluminum fitters using 25 mL round bottom flasks and continuously stirred with a magnetic flea (400 rpm). After 6 h of reaction time at ambient pressure, single reaction flasks were connected to a Schlenk line attached to a vacuum pump V-300 (BÜCHI) connected to a I-300 pressure controller interface (BÜCHI), thereby applying a reduced pressure (15 mbar).

After another 18 h (total reaction time: 24 h), the reaction mixture was recovered in a work-up procedure starting with dissolving the reaction products in MeTHF and removing the biocatalyst through a filtration step using a cotton filter. The solvent was then removed *via* rotary evaporation (R-3, BÜCHI) at 30 °C and then at reduced pressure (6 × 10^−6^ torr) *via* a high vacuum line equipped with a cold trap connected to an oil pump (Edwards RV3 oil filled rotary vane vacuum pump with FL20K front line trap/EMF10), and the polymers were analyzed without further purification steps. The obtained products were prepared in duplicates. Products of enzymatic polycondensation are abbreviated as PE*xy* where *xy* indicates the type of BP monomer and diol used (*e.g.*: PE88 is the product of a polycondensation reaction between C8BPdm and ODO), and the PE in PE*xy* denotes the formed polyester (PE). The reaction products were then analyzed *via* NMR (^1^H-NMR, ^13^C-NMR, and HSQC) (see ESI Fig. S7 to S16†). Further analysis of the reaction products was conducted *via* GPC and TGA (see ESI Table S4, Fig. S28 and S29†).

### Preparation of PLA-based formulations

The bis-pyrrolidone (BPdm) monomers were solvent-mixed with PLA at room temperature. Prior formulation, PLA was dried in a vacuum oven at 30 °C (overnight, at 130 mbar). The mixing was performed using PLA with the addition of 10% of BPdm monomers to the amount of PLA (10% w w^−1^ BP-based/PLA), resulting in a total of about 0.2 g mixture in 10 mL of DCM (final concentration 20 mg mL^−1^). The preparation was conducted in 20 mL glass vials with constant stirring at 1000 rpm for about 2 h. A blank was performed by processing 0.2 g of PLA without adding BPdm monomers.

### Preparation of PLA-based films *via* solvent casting

The polymer films were prepared applying the solvent casting method as follows. The polymer solution was prepared by dissolving 200 mg of polymer formulation as described in the section “Preparation of PLA-based formulations”. The solution was poured into a 54 mm diameter glass Petri dish, and the solvent was evaporated overnight at 30 °C in an oven. To further dry the films, they were then placed in a vacuum oven (Bicasa Vuotomatic 50 Vacuum oven) at 30 °C and 130 mbar for 72 h. Subsequently, the samples were stored in a desiccator over molecular sieves until mechanical testing. The films were weighed prior to mechanical and thermal testing to assure that all solvent had evaporated, and mechanical properties measured are properties exhibited by the system formed in the formulation. The results of the thermal analysis *via* differential scanning calorimetry (DSC) and thermogravimetric analysis (TGA), and mechanical testing are shown in Table 3 and in the ESI (Fig. S30 and S34†). All solvent-cast films were prepared in duplicates.

### Characterization

#### Gas chromatography coupled with a mass spectrometer (GC-MS)

Itaconic-based diester building blocks (C*x*BPdm, described above) subsequently used for polymer synthesis were diluted to 100 ppm with ethyl acetate (EtOAc) in 1.5 mL HPLC vials. Gas chromatography was carried out using a GC-MS solution workstation and software for Shimadzu GC-MS-QP2010 SE series gas chromatograph-mass spectrometers (Kyoto, Japan) connected to a Hichrom HI-5 MS column with a capillary of 30 m × 0.25 mm × 0.25 μm nominal. The injector (AOC-20i Plus Auto Injection) provided a flow rate of 1 mL min^−1^ of He. A heating rate of 25 °C min^−1^ and an initial hold time at 70 °C for 3 minutes were chosen. After reaching the final temperature of 300 °C, the column oven temperature was maintained for 6 minutes. The first segment was set to 70 °C as the column oven temperature and 250 °C as the injection temperature.

#### High-performance liquid chromatography coupled with a mass spectrometer (HPLC-MS)

High-performance liquid chromatography/electrospray mass spectrometry (HPLC-MS) with positive ionization was used to perform a complete scan from 100 to 800 *m*/*z* of the synthesized bis-pyrrolidone compounds. The analytes were separated using an HPLC (1100 series, Agilent Technologies, Palo Alto, CA) equipped with a Phenomenex Synergi Hydro reverse phase column of 150 × 3 mm and 4 μm particle diameter. Mobile phase A was ddH_2_O water with 0.1% formic acid, and mobile phase B consisted of LC/MS grade acetonitrile with 0.1% formic acid. The flow rate was set to 0.5 mL min^−1^, the temperature inside the column was set to 30 °C, and the injection volume was 5 μL. 10 min of post-time run time were used to equilibrate the column. The HPLC was connected to a Microsaic 4000 MiD® equipped with a microflow electrospray ion source (spraychip®). A calibration standard (sodium formate solution 1 mg ml^−1^ in 2-propanol/water 1 : 1) over the range *m*/*z* 50–800 was used to autocalibrate the mass with reference calibration masses (see ESI Table S2†). Spectra were gathered with a count time of 0.207 ms.

#### Nuclear magnetic resonance (NMR)

All ^1^H-NMR, ^13^C-NMR, and HSQC spectra were recorded on a NMR JEOL ECZ-400R/S3 5 mm (resonance frequencies 400 MHz for ^1^H, 100 MHz for ^13^C) equipped with a broadband Royal HFX Probe. The samples (approximately 20 mg) were dissolved in 0.7 mL of CDCl_3_ (99.8% D, containing 0.03% (v/v) TMS). Chemical shifts are given in ppm using the TMS signal as the reference.

The conversion of the monomers for BPdm synthesis (eqn (1)) and polymerization (eqn (2)), reported in Table 2, were calculated from ^1^H-NMR by referring to the integral if the proton signal of terminal groups from the educts and the integral of their counter signal as an incorporated segment of the synthesized structure. Eqn (1) relates the integrated value of the proton signals associated with the different methoxy groups (referred to as O–CH_3_ in eqn (1)) with each other. The proton signals of the methoxy groups of the aspired BPdm are placed in relation to all proton signals associated methoxy groups of DMI (reacted and unreacted).

The percentage of the signal corresponding to the product's methoxy group (7.71 ppm) is calculated relative to the total signals from all methoxy groups, including those of the educt, DMI (7.78 ppm), its regioisomer (dimethyl mesaconate, 7.74 ppm), and the product itself (7.71 ppm). For more details, refer to Fig. S0 in the ESI.†1
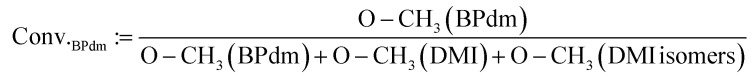


The conversion of the monomers (BPdm and diols) after polymerization reactions was calculated by taking the average of two values which refer each to the integral of the proton signal of terminal groups from the monomers and their counter signal as an incorporated segment of the oligomers. The conversion of the different terminal groups, which after assimilation into the polymer structure build part of the backbone, should be approximately the same. The first value was obtained by comparing the ratio between the methoxy group protons (referred to as –O–CH_3_ in eqn (2)) of the oligoester chain (at 4.11 ppm for the methoxy groups of the oligoester chain) and the initial value of the methoxy groups of the dicarboxylic methyl ester (assumed as constant *k*1 = 6); the second component of the equation focuses on the ratio between the reacted and unreacted diol by referring to the protons belonging to the –C**H**_**2**_–OH of the diol (at 3.72 ppm) and the corresponding signals incorporated into the polyester structure (at 4.11 ppm in eqn (2)).2
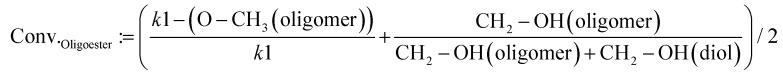


The degree of polymerization (DP) of the products (reported in Table 2) was calculated from ^1^H-NMR by taking the ratio of the present and expected signals using the following definition: let *X* represent the sum of all normalized proton signals associated with the product in the spectrum. *X* is then multiplied by the number of proton signals linked to the methoxy and terminal hydroxy groups of the oligoester, assumed to be constant (*k*2 = 5). The denominator consists of two parts: *Y*, which is the sum of all proton signals theoretically expected for the corresponding repeating units of the oligoester, and the remaining signals from the methoxy group and terminal carbon attached to the alcohol groups of the unreacted diester and diol (referred to as –O–C**H**_**3**_ (diester) at 3.62 ppm and –C**H**_**2**_–OH (diol) at 3.72 ppm in eqn (3)).3
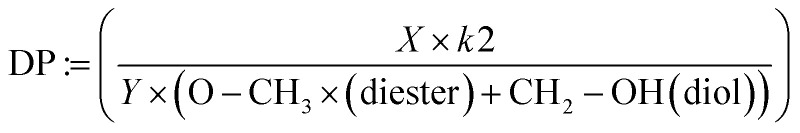


#### Gel permeation chromatography (GPC)

The samples were dissolved in CHCl_3_ at a concentration of 2 mg mL^−1^ and filtered through a cotton filter. Gel permeation chromatography was carried out on an Agilent Technologies HPLC System (Agilent Technologies 1260 Infinity) connected to a 17 369 6.0 mm ID × 40 mm L HHR-H, 5 μm guard column and an 18 055 7.8 mm ID × 300 mm L GMHHR-N, 5 μm TSK gel liquid chromatography column (Tosoh Bioscience, Tessenderlo, Belgium), using CHCl_3_ as the eluent (at a flow rate of 1 mL min^−1^) at 30 °C. An Agilent Technologies G1362A refractive index detector was used for detection. The calibration standards used to calculate the molecular weights of the polymers were purchased from Sigma-Aldrich (linear polystyrene calibration standards ranging from 400 to 2 000 000 Da).

#### Fourier-transform infrared spectroscopy (FT-IR)

Fourier-transform infrared (FT-IR) spectroscopy was performed using a VERTEX 70v FT-IR spectrometer (Bruker Corporation, Boston), in ATR mode, running 128 scans with a resolution of 2 cm^−1^.

#### Thermogravimetric analysis (TGA)

The thermogravimetric analysis (TGA) was performed using a Mettler Toledo “TGA/DSC1 STAR^e^ System®” operating in a temperature range from 30 to 800 °C with a heating rate of 10 °C min^−1^. In the first segment (30–700 °C), a nitrogen flow of 80 mL min^−1^ was used to ensure degradation under inert conditions. Subsequently, the second phase (700–800 °C) was performed under an oxidizing atmosphere by switching to an O_2_ flow of 80 mL min^−1^. All the thermograms were corrected by subtracting the blank curve of the empty crucible obtained under the same analysis conditions. The thermogravimetric curves are shown in Fig. S25 to S28 in the ESI.† The onset degradation temperature (*T*_onset_), temperature of the maximum rate of degradation for the first (*T*_max1_) and second step (*T*_max2_) were determined as shown in Fig. S24 in the ESI.†

#### Differential scanning calorimetry (DSC)

Differential scanning calorimetry (DSC) analysis was performed with a Mettler Toledo “DSC1 STAR^e^ System®” using 40 μL aluminum pans with a singular central perforation of the lid. An empty aluminum pan with a perforated lid served as a reference. The measurements were performed under dry N_2_, with a heating phase from 0 to 200 °C, followed by a cooling phase to −100 °C, and another heating phase up to 200 °C. Both the heating and cooling phases were performed at a rate of 10 °C min^−1^. The scanning calorimetry curves are shown in Fig. S29 to S32 in the ESI.†

#### Mechanical testing (stress–strain)

Strain tests were performed using an Instron mechanical tester (Instron 5565) at a speed of 0.5 mm min^−1^ with an initial gage length of 20 mm. The specimens were cut into 30 × 10 × 0.2 mm^3^ samples from films previously dried in a vacuum oven and then stored in a desiccator, as described in the section Preparation of polymer films *via* solvent casting. Analysis of the tensile strength was performed according to the parameters shown in ESI, Fig. S33.†

## Results and discussion

### Synthesis of itaconic acid-based bis-pyrrolidone

**Fig. 2:**
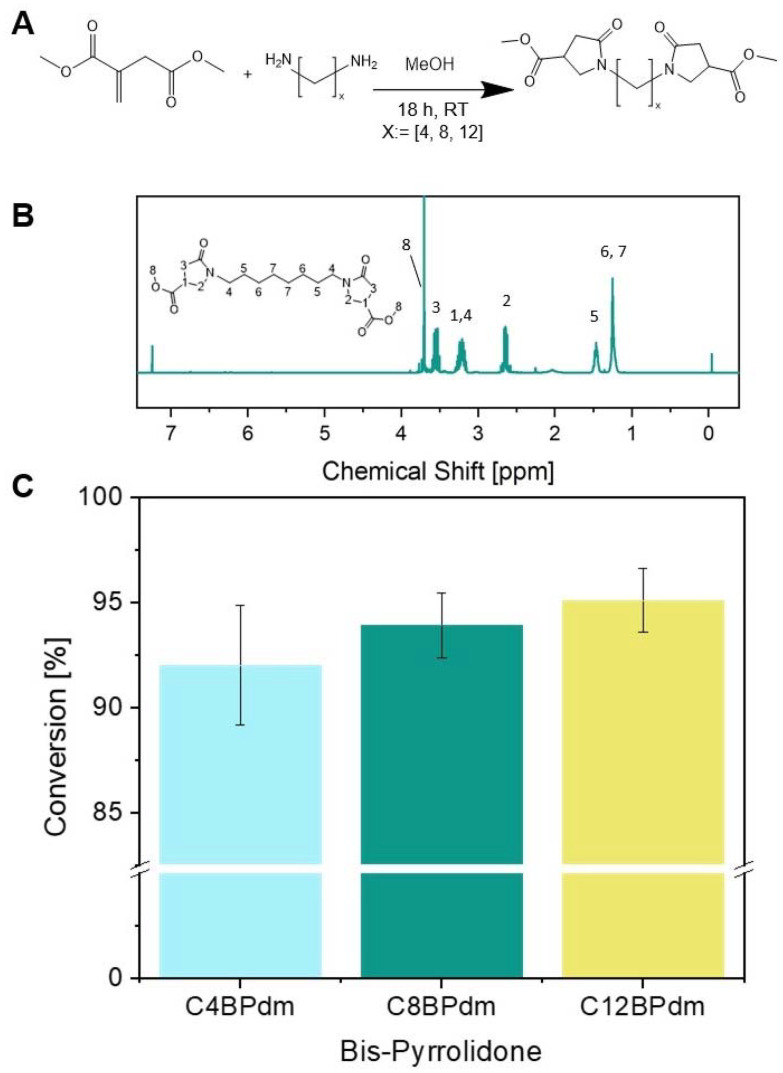
Synthesis of the bis-pyrrolidone (C*x*BPdm) building blocks. (A) The reaction between DMI and diamine spacers with *x*: = [4, 8, and 12] using a catalytic amount of methanol used to prepare the BPdm structures. (B) ^1^H-NMR spectrum of C8BPdm; (C) the raw yield of BPdm structures (calculated using eqn (1)).

The work has been primarily focused on the synthesis of bis-pyrrolidone derivatives with 4, 8 and 12 carbon atom spacers. C4BPdm and C12BPdm were obtained as waxy products while C8BPdm as a viscous oil. FT-IR and ^1^H-NMR analysis were carried out to elucidate and confirm the chemical structure of the synthesized molecules. The ^1^H-NMR spectrum of C8BPdm, given as an example in Fig. 2 and 4 (see also ESI Fig. S1†), showed the proton signals belonging to the central part of the diamino spacer between 1.1 and 1.6 ppm. The proton signals due to the pyrrolidone ring (N–C**H**_**2**_ spacer) are associated with the signals detected from 3.1 to 3.4 ppm. For all three BPdm monomers, the above signal overlapped with that of the –CH moiety inside the ring (bound to the methoxycarbonyl group –COOCH_3_, see ESI Fig. S1 to S5†). The broad signal at around 3.6 is characteristic of the N–C**H**_**2**_ structure inside the pyrrolidone ring, while the –CH_2_ moiety inside the ring resonates at around 2.7 ppm. The signal for the terminal methyl group (O–CH_3_) appeared between 3.7 and 3.8 ppm.

Furthermore, the NMR spectra for C4BPdm and C12BPdm show a comparable set of proton signals and a clear correlation between the different BPdm monomers can be seen when comparing their ^1^H-NMR and ^13^C-NMR spectra (see ESI Fig. S1 and S2†). Undoubtedly, as the chain length of the diamine spacer increases, new signals appear in the low field region of the spectrum corresponding to additional methylenic units of the central alkyl chain (proton signals between 1.20 and 1.36 ppm). The peak's intensity grows further when longer diamino spacers are used.

Since the conversion, calculated by NMR, was high for all BPdm samples (between 93 and 97%, calculated using eqn (1)), the synthesized bis(pyrrolidone) structures can be employed directly for co-polymerization as the obtained purity is comparable to that of other commercial monomers used for polycondensation reactions.

In addition to the data based on the ^1^H-NMR analysis, the purity of the C*x*BPdm compounds was further verified by gas chromatography coupled with a mass spectrometer (GC-MS) (see ESI Fig. S16–S19†) and HPLC-MS (see ESI Fig. S20 and S21†), and the structure was confirmed *via* Fourier-Transform Infrared Spectroscopy (FT-IR) (see ESI Fig. S22†). The ion chromatogram from GC-MS of C4BPdm shows a clear signal eluting after 11.7 min. The signal correlates to a molecule having a *m*/*z* of 340 g mol^−1^, which corresponds to the calculated molecular mass of C4BPdm 340.37 g mol^−1^ (see ESI Table S1†). Running the same thermal program for C8BPdm resulted in a clear peak for a monomolecular ion having a *m*/*z* of 396 g mol^−1^ (see ESI Fig. S16†) and therefore confirming the obtained structure. While GC-MS analysis of C4BPdm and C8BPdm successfully confirmed the presence of structures with the expected molecular weight, no GC-MS signal was detected for C12BPdm. This might be due to the fact that the compound is not sufficiently volatile for GC analysis. Therefore, the sample was analyzed *via* HPLC-MS analysis. The obtained molecular weight for C12BPdm was 452.48 g mol^−1^ (see ESI Fig. S20 and S21†), confirming the structure of the longer BPdm.

The FT-IR spectra of the three BPdm-compounds (see ESI Fig. S22†) confirm the formation of the pyrrolidone ring. A signal around 1490 cm^−1^ corresponds to the vibrational resonances of the tertiary amide group in the pyrrolidone ring. Similarly, the carbonyl stretch of the carboxylic ester (C=O) is observed at around 1750 cm^−1^. Lastly, the presence of the carbonyl stretching in the amide is evidenced by the vibrational signal at around 1670 cm^−1^ for C4BPdm, 1675 cm^−1^ for C8BPdm, and 1680 cm^−1^ for C12BPdm. N–H stretches, which are usually observed as two bands between 3250 to 3400 cm^−1^ for primary amines (such as DAB, DAO, and DADD), were not detected, proving their absence and therefore the high conversion of the molecules.

TGA analysis of the BPdm series was performed to investigate and correlate the thermal stability of the monomers with their chemical structures. By examining the thermograms, different onset degradation temperatures (*T*_onset_) of 317 °C, 323 °C, and 353 °C were found for C4BPdm, C8BPdm and C12BPdm, respectively (see Fig. S25† and Table 1). Notably, these materials displayed a higher thermal stability when compared to their carboxylic acid counterparts studied in other works, whose *T*_onset_ is around 270 °C.^28,29^ This increase was presumably accounted for the presence of methyl ester which possibly hinders the first mass loss step observed for bis-pyrrolidone carboxylic acids, that occurs immediately after 270 °C, probably ascribed to the decarboxylation of the pyrrolidone rings during heating. Additionally, it was observed that *T*_onset_ in the BPdm series increased with the number of methylene units of the spacer, *i.e.*, the aliphatic saturated fraction of the molecule, also evidencing the effect of this moiety on the thermal stability of the compounds. By observing the DTG thermograms of BPdm (Fig. S25B, ESI†) a two-step degradation for all the compounds was evidenced. Specifically, the first degradation step, characterized by a lower temperature, between 379 °C and 411 °C (*T*_max1_) was associated with a higher mass loss, while the second one, occurring between 438 and 467 °C (*T*_max2_), with a lower one. As previously observed for *T*_onset_, the temperature of these thermal events was also found to be susceptible to the length of the central aliphatic spacer (Table 1). Analogous results can be found by observing the thermograms reported by Roy *et al.* for bis-pyrrolidone carboxylic acids.^26^ Also, in this case, it can be observed that an increase in the central alkyl fraction of bis-pyrrolidones, influenced the degradation rate of these compounds, modifying and shifting the mass loss curve to higher temperatures, increasing their thermal stability. However, a direct comparison is not possible as, to the best of our knowledge, the thermal stability of these compounds has not yet been studied in detail.

**Table 1:** GC-MS, HPLC-MS, DSC, and TGA analysis results of BPdm synthesized in this work. The weight of the molecule (*M*_0_) was calculated based on the relative structure and the theoretical mass-to-charge ratio (*m*/*z*_(theo)_). They were confirmed from GC-MS and HPLC-MS (*m*/*z*_(exp)_). The data for the glass transition temperature (*T*_g_), obtained from the DSC analysis (performed at a 10 °C min^−1^ heating/cooling rate), was taken from the second heating cycle

Sample*	*M*_0_ [g mol^−1^]	*m*/*z*_(theo)_	*m*/*z*_(exp)_	*T*_g_ c [°C]	*T*_onset_ d [°C]	*T*_max1_ d [°C]	*T*_max2_ d [°C]
C4BPdm	340.37	340.16	340.0^a^	−32	317	379	438
C8BPdm	396.48	396.23	396.0^a^	−44	326	394	452
C12BPdm	452.58	452.26	453.0^b^	−25	353	411	467

a Calculated from GC-MS analysis.

b Calculated from HPLC-MS analysis.

c Calculated from DSC analysis.

d Calculated from TGA (see also ESI Fig. S24†).

The DSC analysis of BPdm showed a different behavior for the three examined monomers. For C4BPdm and C8BPdm, the complete absence of crystallization and melting peaks in the cooling and second heating thermograms indicates that these monomers cannot crystallize under the conditions of the thermal program used (see ESI Fig. S29†). In contrast, crystallization and melting peaks were observed in the thermogram of C12BPdm, at 24 °C and 39 °C, respectively. The presence of a double melting peak in the second heating suggests the formation of imperfect crystals (see ESI Fig. S29B†). A direct comparison with the literature is difficult since BPdm with aliphatic C4, C8 and C12 spacers, have not yet been employed in any similar work. The only direct comparison can be with the free carboxylic acids studied by Roy *et al.* which are all able to crystallize, facilitated by their ability to form intermolecular hydrogen bonding.^19^ To explain these observations, *i.e.* the inability of C4BPdm and C8BPdm to crystallize, it is therefore appropriate to consider the effects caused by the different molecular structures, since the presence of methyl would impair the ability to form a hydrogen bond and introduce an additional steric hindrance affecting the crystallization process. The importance of this hydrogen bond for thermal properties is highlighted in Roy's work by the observation that the *T*_g_ decreases as the aliphatic chain increases, *i.e.*, as the concentration of –COOH groups per unit mass decreases.^19^ Considering the complete absence of the hydrogen bond, the crystallinity observed for C12BPdm could be related to the longer aliphatic chain and therefore less interference by the pyrrolidone rings. Therefore, C12BPdm can pack more easily to form a crystalline phase. These observations can also be explained presumably by the fact that the steric configuration of the carbon bearing the carboxymethyl group is random, resulting in a diastereoisomeric mixture for each compound. This characteristic could hinder the packing of BPdm molecules to form the crystalline phase, especially for monomers where the aliphatic moiety, capable of favoring the packing and thus the crystallization of the molecules, is not predominant.

### Enzymatic polymer synthesis of bis-pyrrolidone-based compounds

**Fig. 3:**
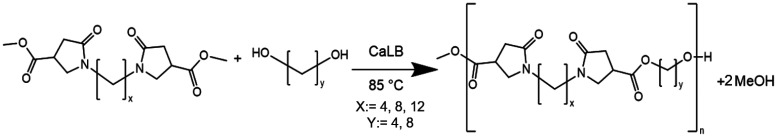
Reaction scheme of BPdm-based polycondensation. The reaction between BPdm structures spacers with *x*: = [4, 8, 12] and a potentially bio-based diol with *y*: = [4, 8] in a solventless enzymatically catalyzed reaction formed the BPdm-based oligomers. After 6 h at ambient pressure a further 18 h of reaction time at reduced pressure were applied.

The following part of the work focused on the enzyme-catalyzed polycondensation of BPdm compounds with two linear aliphatic biobased diols, 1,4-butanediol (BDO) and 1,8-octanediol (ODO). Lipase B from *Candida antarctica* (CaLB) in its immobilized form, known as Novozym® 435, was used as a biocatalyst for all of the polycondensation processes described in this section (Fig. 3). All synthesized BPdm-based oligomers, obtained in the form of colorless to yellow, transparent viscous liquids, were analyzed *via* NMR (see ESI Fig. S6 to S15†), FT-IR (see ESI Fig. S23†), and GPC analysis (see ESI Table S4†). The conversion of monomers was calculated from ^1^H-NMR and was on average 86% (see also Table 2).

**Table 2:** GPC, DSC and TGA analysis of the oligoesters synthesized in this work. The bis-pyrrolidone structures were reacted with two different diols (BDO and ODO) using a solventless-enzymatic polycondensation approach

Sample	*M*_0_ [g mol^−1^]	*M*_n_ a [g mol^−1^]	*M*_w_ a [g mol^−1^]	*Đ* a	DP^b^	*T*_g_ c [°C]	*T*_onset_ d [°C]	*T*_max1_ d [°C]	*T*_max2_ d [°C]	Conv.^e^ [%]
PE44	366.40	800	1000	1.21	2	−2	365	396	448	72
PE48	422.51	2100	3800	1.79	5	−16	391	415	458	85
PE84	422.51	1400	2600	1.94	3	−14	387	413	467	84
PE88	478.62	1300	3000	2.23	3	−25	393	417	472	82
PE124	478.61	2400	4900	2.04	5	−23	384	416	471	92
PE128	534.72	3300	6500	1.98	6	−29	397	416	477	98

a Calculated from GPC analysis.

b Calculated *via M*_n_/*M*_0_.

c Calculated from DSC analysis.

d Calculated from TGA analysis.

e Yield of the isolated product, calculated from ^1^H-NMR using eqn (2).

The measurements of the molecular weights (*M*_n_ and *M*_w_) of the reaction products (Table 2) indicated that oligomers formed are characterized by *M*_n_ from 800 g mol^−1^ to 3300 g mol^−1^. The rather low molecular weights of the synthesized polyesters (thus in the following referred to as oligomers and oligoesters) are most likely due to the chemical nature of the BPdm compounds used, which, containing pyrrolidone rings, represent a rather bulky substrate for the enzyme and exhibit greater steric hindrance and lower chain flexibility than the natural aliphatic substrates. It is likely that BPdm binds less effectively to the enzyme's active site and the pocket around it than the starting molecule DMI. Indeed, the enzyme is known for its high activity and specificity towards primary and secondary alcohols, although molecular modeling and crystallographic studies showed that the binding pocket associated with the active center is sterically restrictive.^28,29^ It was reported that CaLB could only convert fatty acids having different chain lengths, preferably between C6 and C12.^35^ However, catalytic activities of CaLB variants reported in the literature showed that the engineering of CaLB for the hydrolysis of bulky carboxylic acid esters may lead to a shift in the activity toward sterically demanding acyl substrates.^36,37^ The effect of varying the alkylene chain length of diols and diacids on the molecular weight distribution and the polymer structure was assessed in several studies using a series of diacids or diesters and diols polymerized in solution and in bulk.^38,39^ For example, Mahapatro *et al.* reacted succinic, glutaric, adipic, and sebacic acid with 1,4-butanediol, 1,6-hexanediol, and 1,8-octanediol and found that reactions involving monomers having longer alkylene chain lengths (for both diacids and diols) resulted in higher reactivity than reactions of shorter chain-length monomers.^39^ On the other hand, it is known that lipases avoid the formation of complicated structures or cross-linking reactions to a considerable extent due to steric hindrance at the active site.^40^ CaLB-catalyzed polymerizations are reported to process slowly when the 5-hydroxy of dimethyl 5-hydroxyisophthalate monomers was alkylated and had a chain length of C5 or longer. According to Gross *et al.* this revealed how bulky pendant groups along chains induce steric constraints at the CaLB active site, slowing down further chain formation.^40^ This further supports the hypothesized effect of BPdm monomers during polymerization.

**Fig. 4:**
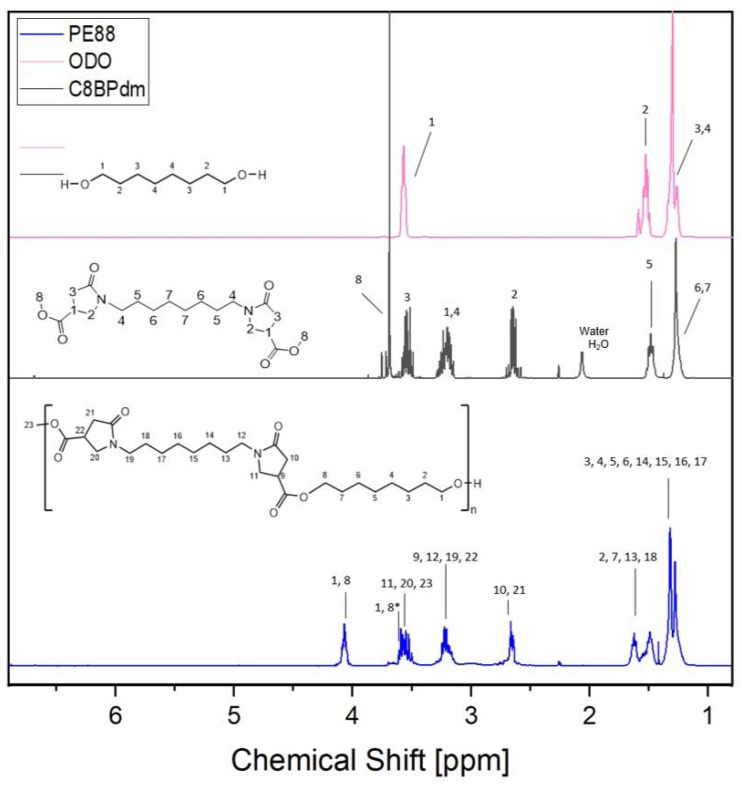
^1^H-NMR spectra of polymer PE88 (blue) and monomers C8BPdm (black) and ODO (pink). The reported spectrum clearly shows the formation of the polyester as the –OC**H**_3_ signal (8 black) of the used C8BPdm monomer had a greatly reduced intensity in the final product while the intensity of the signal at 4.1 ppm (–CH_2_–C**H**_2_–O–C=O, 1,8 blue) increases.

The NMR spectra showed the proton signals described for the corresponding BPdm monomers (spacer (–C**H**_**2**_)_*x*_: 1.1–1.6 ppm; N–C**H**_**2**_ spacer & –CH in the ring: 3.1–3.4 ppm; N–C**H**_**2**_ in the ring: 3.6 ppm; –C**H**_**2**_– in the ring: 2.7 ppm; O–C**H**_**3**_: 3.7–3.8 ppm, see black and blue signals in Fig. 4) with additional signals between 1.2 and 1.8 ppm, overlying the aliphatic signals from the BPdm monomers and corresponding to the aliphatic chain introduced by the diol. Moreover, a new signal around 4.1 ppm marks the external –CH_2_ of the diol (–CH_2_–C**H**_**2**_–O–C=O, see the blue signal in Fig. 4) close to the newly formed ester bond.

It is worth mentioning the relationship between the signals at 4.1 ppm and 3.7–3.8 ppm: the longer the polyester chain, the weaker the O–C**H**_**3**_ signal and the stronger the signal for the (R–C(=O)–O–C**H**_**2**_–R′) appear in relation to each other. All BPdm-based oligoesters were also analyzed *via*^13^C- and HSQC-NMR (see ESI Fig. S7–S15†) for ensuring a complete characterization of the obtained structures. Analogously to the ^1^H-NMR, the ^13^C-NMR showed signals originating from the respective assimilated BPdm monomer (–**C**=O ester: 172.6–172.8 ppm; –N–**C**=O: 173.8–173.9 ppm; –N–**C**H_2_– ring: 49.1–49.4 ppm; –**C**H ring: 36.0–36.5 ppm; –N–**C**H_2_– spacer: 42.2–43.1 ppm; –**C**H_2_–: 34.5–34.8 ppm) and together with these also the ester bond at 66 ppm, confirming the incorporation of the BPdm structure in a polyester chain. Again, the HSQC analysis verified the deduced correlations. Furthermore, the recorded NMR spectra are very similar to those reported by Qi *et al.*^41^ in the preparation of some polylactam esters, confirming the assumption that similar structures were obtained in the polycondensation reactions performed in this work.

All synthesized C*x*BPdm-based oligomers showed similar ^1^H-NMR spectra, clearly demonstrating the formation of the expected polyester chains (see ESI Fig. S6†), but differed in the proton region of the aliphatic chain, which can be attributed to the use of the different diols (BDO and ODO) and diamines (used during the C*x*BPdm synthesis) (ESI, Fig. S6†). The proton signal at 4.1 ppm of the methylenic unit (–CH_2_–C**H**_**2**_–O–C=O) and the sum of the areas of the protons of the non-esterified –O–C**H**_3_ groups of the C*x*BPdm at approximately 3.65 ppm were used to calculate the conversion rates (Fig. 4).

The DSC traces of the developed oligomers showed no crystallization/melting peaks, which indicates that they are amorphous (see ESI Fig. S30 and S31†). Certainly, as previously reported, it is possible to hypothesize that BP-containing compounds are bulky enough to hamper the polymer structuring.^28,41^ Concerning the glass transition temperature (*T*_g_), values ranging from −2 °C to −29 °C were determined (Table 2). It is worth mentioning that polyesters based on aliphatic BP monomers generally have a higher *T*_g_ than those found in our systems and additionally in the literature reported *T*_g_ values are highly variable. For example, Qi *et al.* obtained a *T*_g_ of 24 °C for C2-BP + 1,6-hexanediol and 39 °C for C2-BP + 1,4-butanediol, while in the work of Noordzij *et al.*, a *T*_g_ of 5.7 °C was reported for C2-BP + 1,6-hexanediol. The differences in *T*_g_ found in our work and in the literature can be explained by the fact that this parameter depends on the monomer used as well as on the specific characteristics of the final polymer, such as the molecular weight. Furthermore, it was found that the *T*_g_ of BP-based oligoesters decreased significantly with increasing diol and/or diamine spacer length. This interesting result shows that it is possible to tune the specific properties of the polymer by changing the monomer features. An explanation for the different behavior displayed by the produced oligomers can be found by considering what was reported in the previous work by Qi *et al.* where monomers similar to here described C*x*BPdm were synthesized and used in polymerization reactions.^41^ The monomer, ethylene bis(pyrrolidone carboxylic acid) (EBPC), was observed to lead to a polymer which was unable to crystallize.^41^ In another study, Noordzij *et al.* showed that long chain aliphatic or rigid diamine structures in BP monomers can promote structuration and crystallization processes leading to the formation of semicrystalline polyesters by copolymerization with poly(hexamethylene sebacate).^19^ Based on these observations, it can be argued that the aliphatic structure can positively influence the development of crystallinity in these oligomers.

The thermal properties of the developed oligomers were also evaluated by TGA analysis. Apart from sample PE44, whose *T*_onset_ was 365 °C, the other oligomers showed a *T*_onset_ of approximately 390 °C, with only minor differences. Furthermore, it is worth noting that the degradation of the oligoesters occurs in two steps, as was also observed for the starting monomers. As with *T*_onset_, a lower *T*_max1_ was also observed for PE44, which can be attributed to the lower thermal stability of the starting monomer. However, when comparing the thermal behavior of the monomers with that of the corresponding oligoesters, the latter systems are generally characterized by higher *T*_onset_ and *T*_max1_ values, which are to be expected for the increase in molecular weight during the transition from a monomer to a macromolecular system.

### Use of bis-pyrrolidone-based compounds as PLA additives

**Fig. 5:**
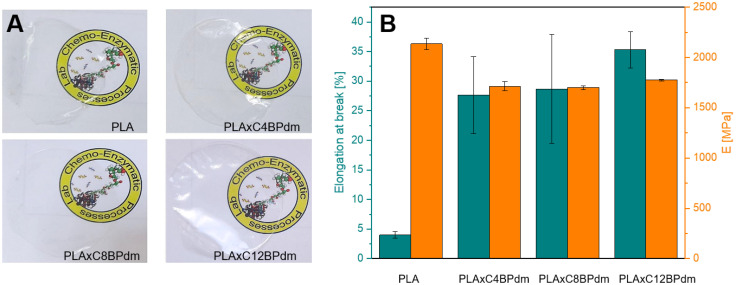
Mechanical characterization of the obtained blended PLA films. (A) Photos of PLA and PLA BPdm-containing films: neat PLA, PLAxC4BPdm, PLAxC8BPdm, and PLAxC12BPdm. (B) Elongation at break (*ε*_break_) and Young's modulus (*E*) from the stress–strain test of PLA and PLA BPdm-containing films. Error bars represent the corresponding standard error.

To evaluate the potential application of BPdm-based compounds prepared in this work, C4BPdm, C8BPdm, and C12BPdm were used as additives in the formulations of polymer films based on poly(lactic acid) (PLA), a cost-competitive bio-based polymer with existing large-scale production facilities, which suffers from excessive brittleness.^42,43^ The idea of using our compounds as additives for PLA is based on the evaluation of the specific structures which, as already highlighted, can increase the free volume of the polymer matrix.^28^ Solvent casted films were prepared and used for mechanical and thermal analysis (see Fig. 5, as well as Fig. S28 and S32 in the ESI†), the results of which are reported in Table 3.

**Table 3:** Thermal and mechanical analysis of neat PLA and the PLA formulations based on 10% w w^−1^ BPdm

Sample code	*T*_g_ a [°C]	*T*_onset_ b [°C]	*T*_max_ c [°C]	*E* d [MPa]	*ε*_break_ d [%]
PLA	59	308	358	2135 ± 55	4 ± 1
PLAxC4BPdm	47	305	359	1715 ± 45	28 ± 6
PLAxC8BPdm	47	310	358	1700 ± 20	29 ± 9
PLAxC12BPdm	43	304	354	1775 ± 10	35 ± 3

a Calculated from DSC analysis of the second heating cycle.

b Calculated from TGA analysis.

c Calculated from DTG analysis.

d Reported as mean ± standard error.

Films containing the BPdm compounds lead to homogeneous, transparent films (Fig. 5A), the PLAxC*x*BPdm films being optically undistinguished from neat PLA films. The films are transparent and do not exhibit any significant changes in coloration, which indicate that PLA and the additive are fully miscible. All the resulting films PLAxC*x*BPdm exhibited a lower *T*_g_ than the neat PLA, which was characterized by a *T*_g_ of 59 °C. The *T*_g_ in amorphous PLA ranges usually from 50 to 60 °C. Below 50 °C PLA exhibits good tensile strength but high brittleness. The use of a plasticizer reduces the *T*_g_, increases plastic elongation PLA chain mobility and decreases brittleness.^42,43^ In particular, the difference in *T*_g_ observed here was more than 10 °C and became as high as 16 °C for PLAxC12BPdm (Table 3). This phenomenon can be ascribed to a plasticizing effect, which is slightly influenced by the chemical structure of the additive, in terms of the alkyl spacer length, being comparable for the three compounds examined. Specifically, this effect can be traced back to the bulky structure of carboxymethyl-bearing pyrrolidone rings of BPdm which can therefore lead, like other common plasticizers, to an increased spacing between PLA chains, increasing the elongation at break by reducing inter-chain interactions and facilitating the chain mobility. Moreover, a nucleating effect generated by the presence of these compounds in the PLA matrix was observed. Specifically, this can be evidenced by a significant increase in the cold crystallization process, which is particularly noticeable for the PLAxC8BPdm and PLAxC12BPdm samples. As already described by other authors, this can be attributed to the formation of clusters, at the molecular scale, between these compounds and the PLA chains, which can lead to an improvement in the short-range structural order, consequently promoting the process of crystalline nuclei formation. From the results of TGA characterization (Fig. S28†), it can be deduced that the thermal degradation behavior was not significantly affected by the addition of the synthesized BP compounds to the polymer matrix, with *T*_onset_ and *T*_max_ of PLA being 308 °C and 358 °C, respectively, while *T*_onset_ and *T*_max1_ of PLAxCxBPdm are in the range of 304–310 °C and 354–359 °C, respectively.

Fig. 5B and Table 3 show the elongation at break (*ε*_break_) and Young's modulus (*E*) of the neat PLA film and that of the systems prepared by adding the synthesized BPdm compounds to the polymer matrix. In fact, at room temperature, PLA is known to be a glassy polymer, with a low elongation at break, of ∼4%.^44–47^ Interestingly, the addition of BPdm compounds leads to a significant increase of the above parameter, reaching 28% for PLAxC4BPdm, 29% for PLAxC8BPdm and 35% for PLAxC12BPdm, a phenomenon which, as previously highlighted, can be ascribed to the peculiar bulky structure of the bis-pyrrolidone-based additives.^28,41^ It is worth understanding that the increase in *ε*_break_ is not accompanied by a likewise drastic change in modulus, E decreasing from *ca*. 2100 MPa for the neat PLA to *ca*. 1700 MPa in the samples with the additives.

In comparison to other innovative PLA blend films these parameters strike as significant. Even though no use of bispyrrolidone systems for plasticization of PLA is reported, similar small molecule plasticizers are described in the literature. One pyrrolidone-containing example is *N*-methyl-2-pyrrolidone (NMP), an organic compound widely used in the petrochemical and polymer industries. Liu *et al.* reported in Mar 2022 the preparation of PLA films with 10% w w^−1^ NMP added in a nonsolvent-induced phase separation method. The films showed uniform porous structures and improved thermal stability.^48^

Byun *et al.*^49^ reported an elongation at break of ∼6% for neat PLA, ∼9% for a PLA film with butylated hydroxytoluene (BHT), and polyethylene glycol 400 (PEG 400), and ∼15% for a PLA film with α-tocopherol, BHT, and PEG 400 (ABP-PLA film) in the machine direction.^49^

The reported results indicate a comparable improvement to the BPdm systems used in our PLA films despite the differences in plasticizer (11% w w^−1^ of plasticizer mix *vs*. 10% w w^−1^ of C*x*BPdm), scale (2.78 kg *vs*. 0.2 g), and film preparation (Byun *et al.* used a cast film extruder *vs*. films obtained *via* solvent casting). Further differences are attributed to the distinct molecular interactions and dispersion of the plasticizer within the PLA matrix.

In addition, the results demonstrate that the specific structure of the additive has no appreciable effect on increasing the ductility of the polymer matrix, which is similar for all three blended systems, suggesting that the pyrrolidone ring plays a more important role than the length of the central aliphatic spacer. These properties, as well as the transparency of the films and their thermal stability, which are not affected by the incorporation of the additives, make the developed systems promising compounds in various areas such as packaging.

## Conclusions

In this work, an effective approach has been developed for the valorization of itaconic acid, which is a promising molecule obtained from renewable sources. Dimethyl itaconate-derived bis-pyrrolidone compounds (C*x*BPdm) were prepared using a scalable and environmentally friendly process, achieving conversions between 93 to 97% without the need for additional purification procedures. The work demonstrated that the synthesized compounds can be successfully used both as monomers in polyester synthesis, exploiting biomass-derived diols as comonomers as well as additives in PLA-based formulations. As all synthesized BPdm compounds were liquid under the conditions applied during the polymerization process, mild and solvent-free enzymatic catalysis could be developed. Oligoesters having molecular weights ranging from 1000 g mol^−1^ to 6500 g mol^−1^ and low dispersity values (<2) were obtained using the bio-based solvent MeTHF in the final workup procedure. Finally, the use of BPdm in PLA-based formulations, which represents a completely new application of bis-pyrrolidone-based compounds, led to an up to 8-fold increase in the elongation at break of the polymer matrix. The bio-derivable nature of the starting resources together with the low environmental impact of the applied synthesis methods as well as the properties make the prepared compounds promising materials for the development of sustainable polymer systems and additives.

## Author contributions

N.S. carried out the monomer synthesis and enzymatic polymerization. N.S. and G.D. prepared the blends, conducted the thermal analysis of the materials, and carried out the stress–strain experiments. N.S. carried out the NMR analysis. N.S. and A.P. wrote the manuscript. The manuscript was revised by all authors. A.T., O.M. and A.P. supervised the work. A.P. acquired the funding.

## Data availability

The data supporting this article have been included as part of the ESI.†

## Conflicts of interest

The authors declare no competing financial interest.

## Supplementary Material

GC-027-D4GC04951A-s001
