# Supplementary material for: Bis-pyrrolidone structures as versatile building blocks for the synthesis of bio-based polyesters and for the preparation of additives
Source: Green Chem. 2025 Jan 3;27(7):1984–96. doi: 10.1039/d4gc04951a (PMC11736262; doi:10.1039/d4gc04951a)
Supplement: GC-027-D4GC04951A-s001 [file GC-027-D4GC04951A-s001.pdf]

This journal is © The Royal Society of Chemistry 2025

Electronic Supplementary Information

Bis-pyrrolidone structures as versatile building blocks for the synthesis of bio-based polyesters and for the preparation of additives

Nele Schulte[1], Giacomo Damonte[1], Valeria Marisa Rocca[1], Anamaria Todea[2], Orietta Monticelli[1], Alessandro Pellis[1,*]

[1] Università degli Studi di Genova, Dipartimento di Chimica e Chimica Industriale, via Dodecaneso 31, 16146, Genova, Italy

[2] Faculty of Industrial Chemistry and Environmental Engineering, Polytechnic University of Timișoara, Carol Telbisz 6, 300001 Timișoara, Romania

* Correspondence to: Dr. Alessandro Pellis, email: alessandro.pellis@unige.it

Table of Contents

Electronic Supplementary Information I
1. NMR Analysis II
   Analysis of Monomers II
   Analysis of Polymers VI
2. GC-MS Analysis XI
3. HPLC-MS Analysis XII
4. FT-IR Analysis XV
   Analysis of Monomers XV
   Analysis of Polymers XVI
5. TGA Analysis XVII
   Analysis of Monomers XVII
   Analysis of Polymers XVIII
   Analysis of PLA Formulations XIX
6. DSC Analysis XIX
   Analysis of Monomers XIX
   Analysis of Polymers XX
   Analysis of PLA Formulations XXI
7. GPC Analysis XXI

Notes:

(1 The obtained BP-dm monomers are abbreviated as C*x*BPdm with x denoting the type of diamine used (*e.g.*: BP-dm with 1,8-diaminooctane: C8BPdm)

(2 The obtained polymers are abbreviated as PE*xy* with *xy* denoting the type of itaconic-based monomer and diol used (*e.g.*: PE88 being the product of a polycondensation reaction between C8BPdm and 1,8-octanediol).

(3 Polymer formulations are abbreviated as PLAxC*x*BPdm with C*x*BPdm as defined above (*e.g.*: PLAxC12BPdm being the product of blending PLA with 10% w w^{-1} C12BPdm).

1. NMR Analysis

Analysis of Monomers

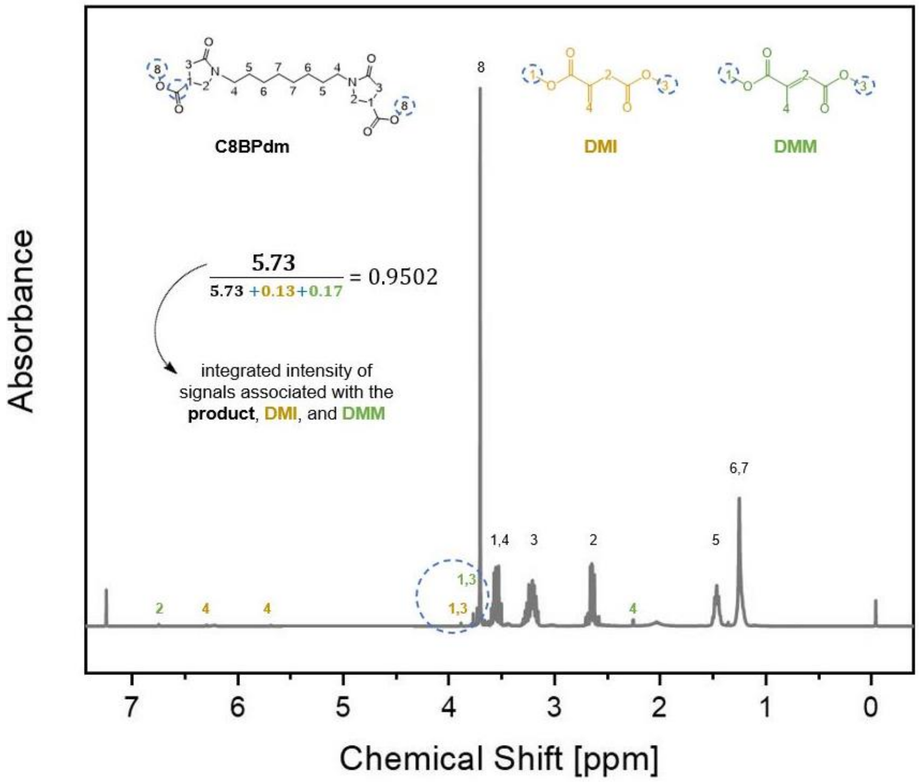

Figure S0. ^{1}H-NMR spectrum of C8BPdm. The conversion of the monomers was calculated to be 95%. The integrated intensity of signals associated with DMI (annotated in yellow) as the educt and dimethyl mesaconate (DMM, green), as its regioisomer, are set in relation to the product (black) as described by **Equation 1**.

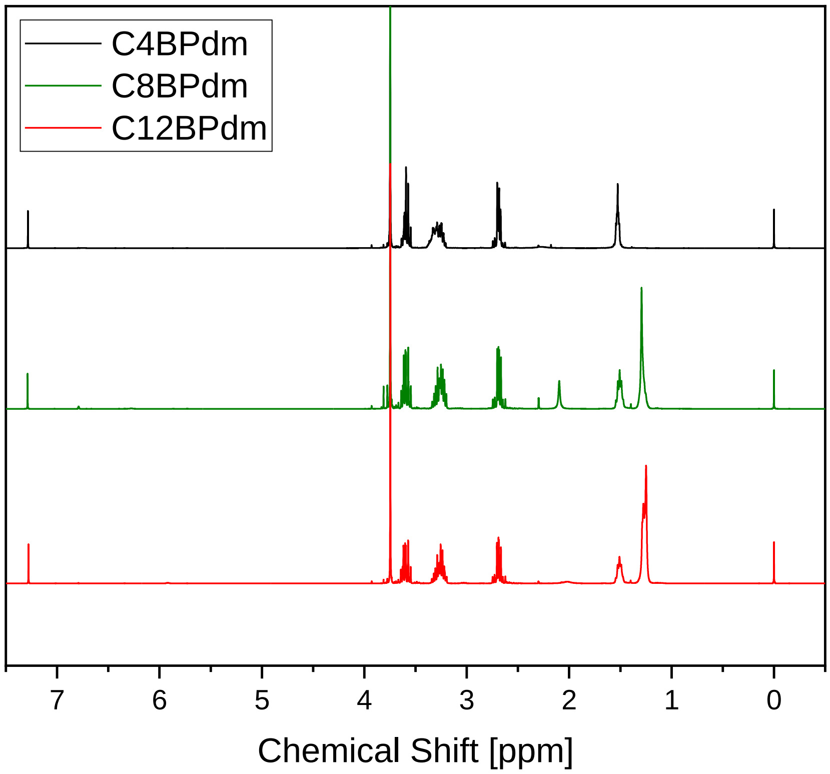

Figure S1. ^{1}H-NMR of C4BPdm, C8BPdm, C12BPdm. The peak around 2 ppm for C8Pdm is free OH originating from moisture in the monomer. C8Pdm is liquid and retains moisture well while C4BPdm and C12BPdm were obtained as waxy substances.

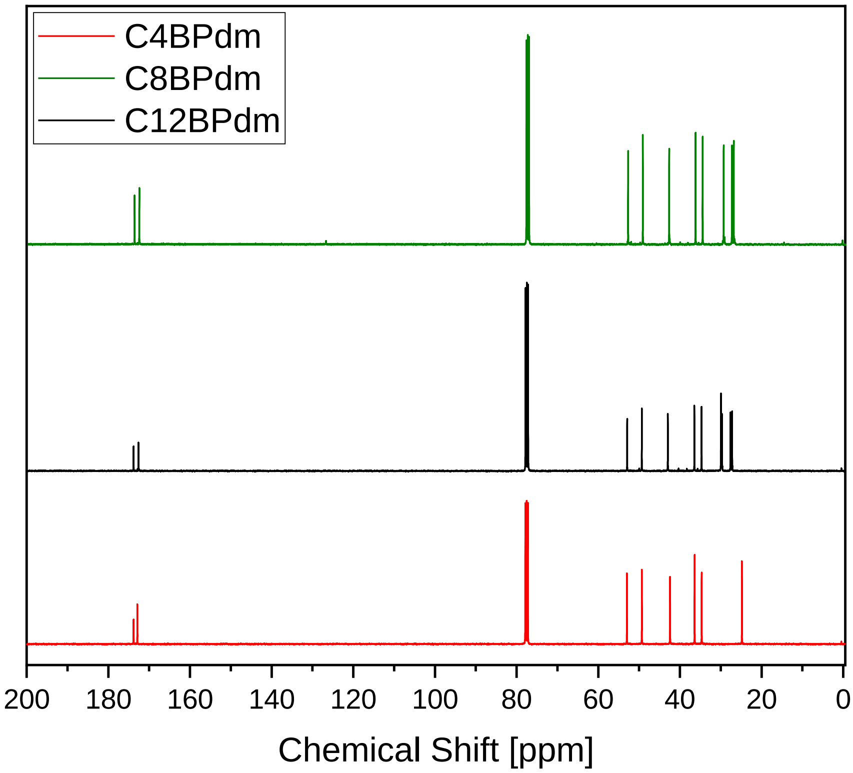

Figure S2. ^{13}C-NMR of C4BPdm, C8BPdm, C12BPdm.

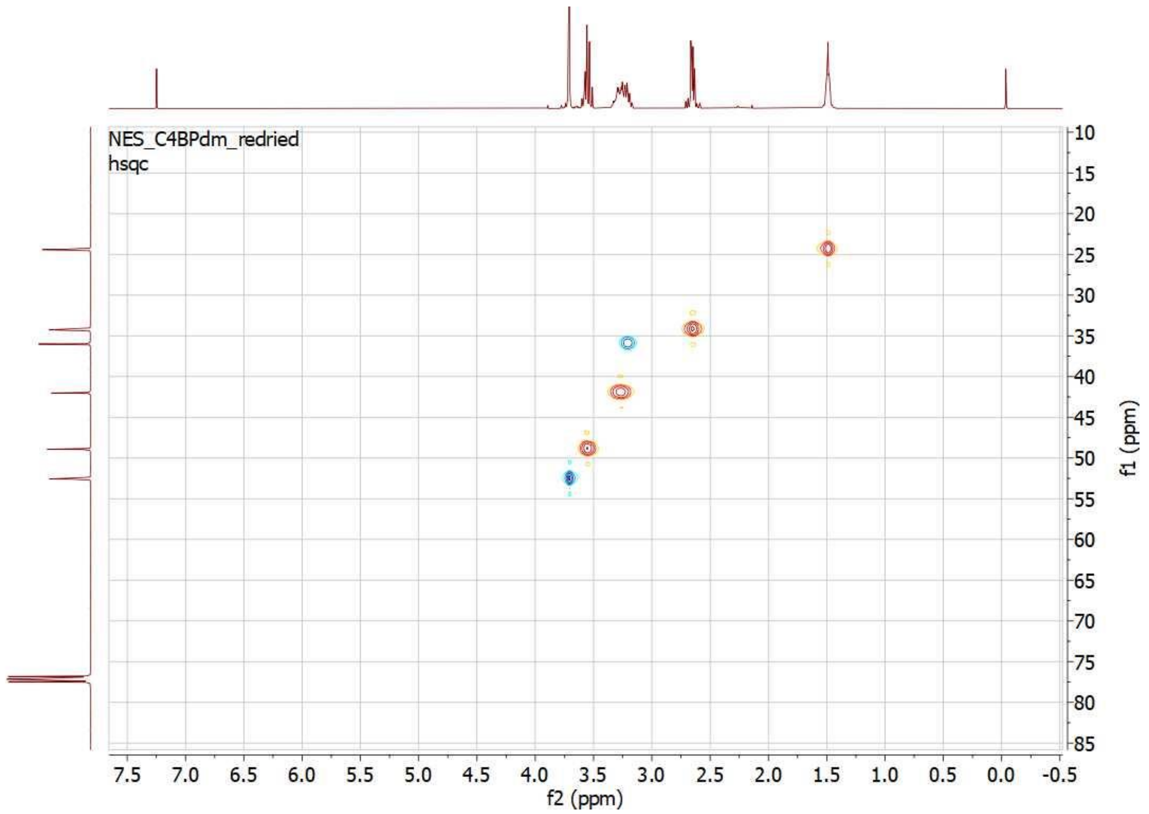

Figure S3. HSQC (^{1}H-^{13}C APT)-NMR of C4BPdm.

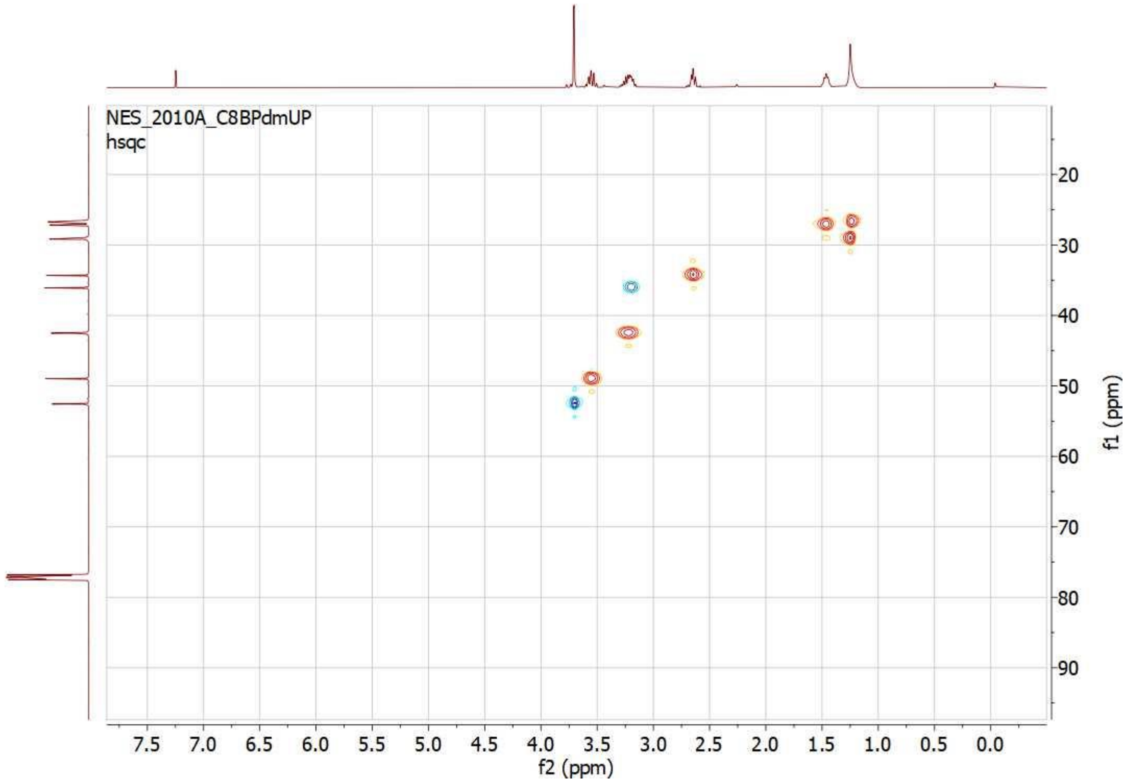

Figure S4. HSQC (^{1}H-^{13}C APT)-NMR of C8BPdm.

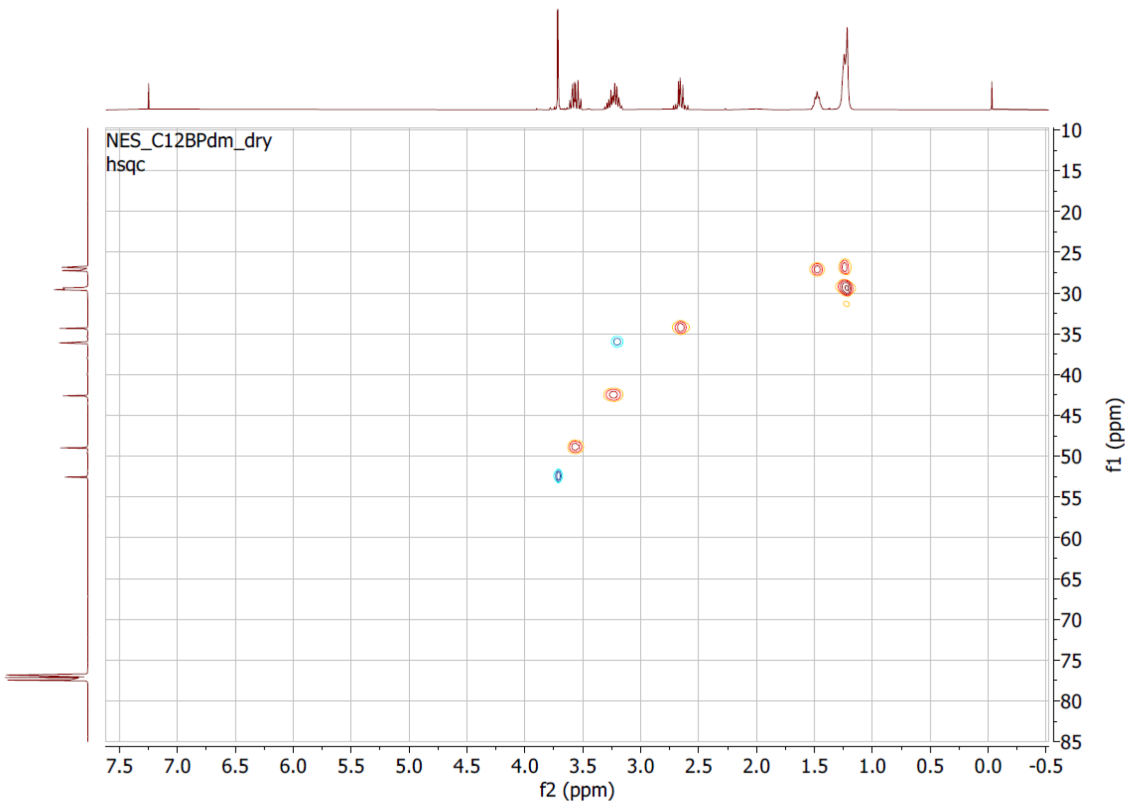

Figure S5. HSQC (^{1}H-^{13}C APT)-NMR of C12BPdm.

Analysis of Polymers

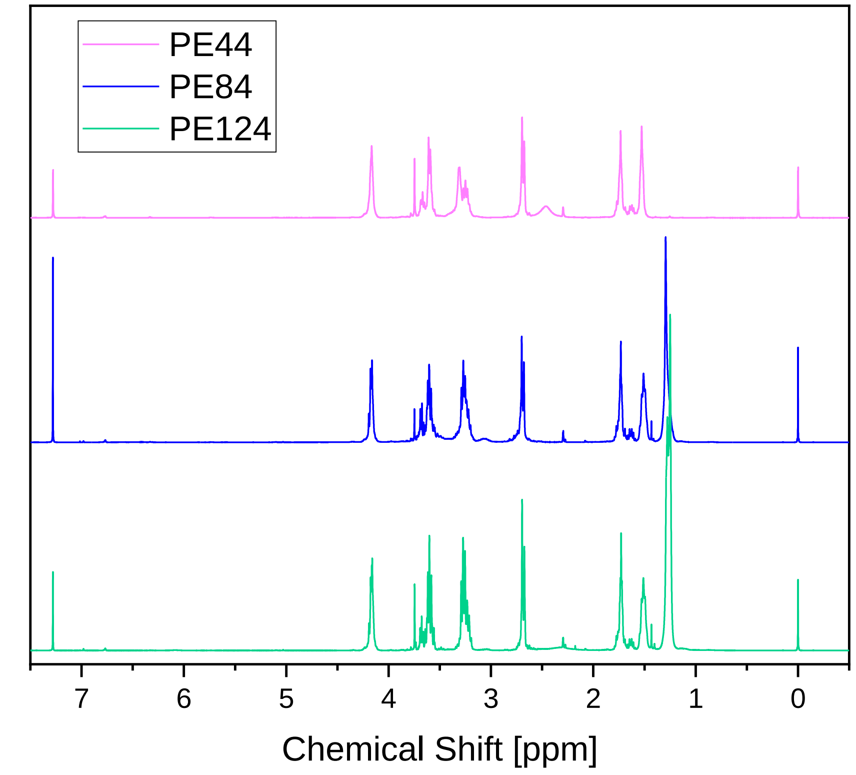

Figure S6. ^{1}H-NMR of PE44, PE84, PE124.

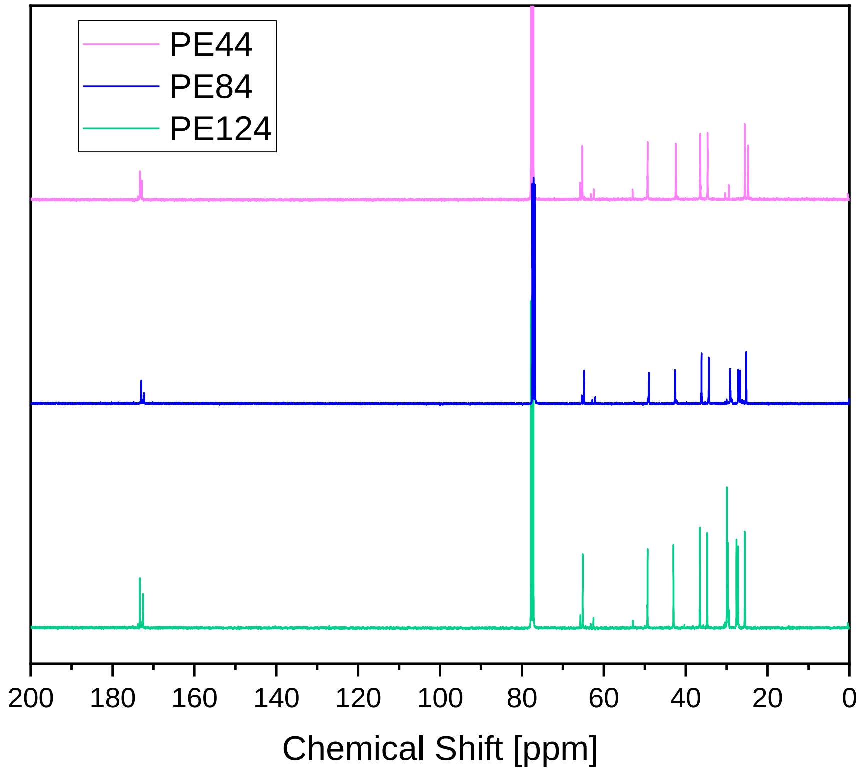

Figure S7. ^{13}C-NMR of PE44, PE84, PE124.

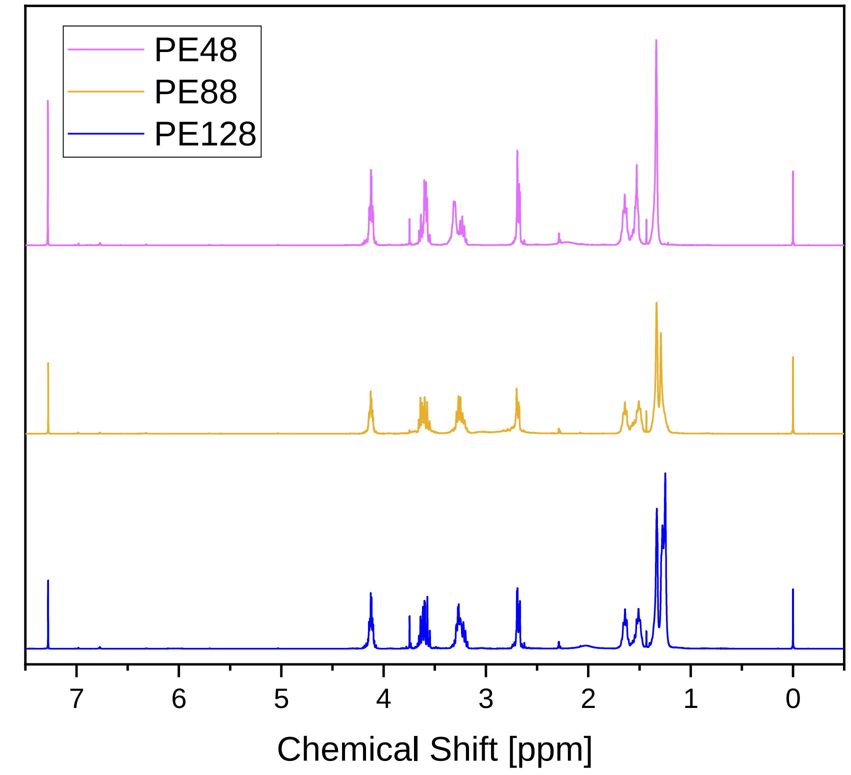

Figure S8. ^{1}H-NMR of PE48, PE88, PE128.

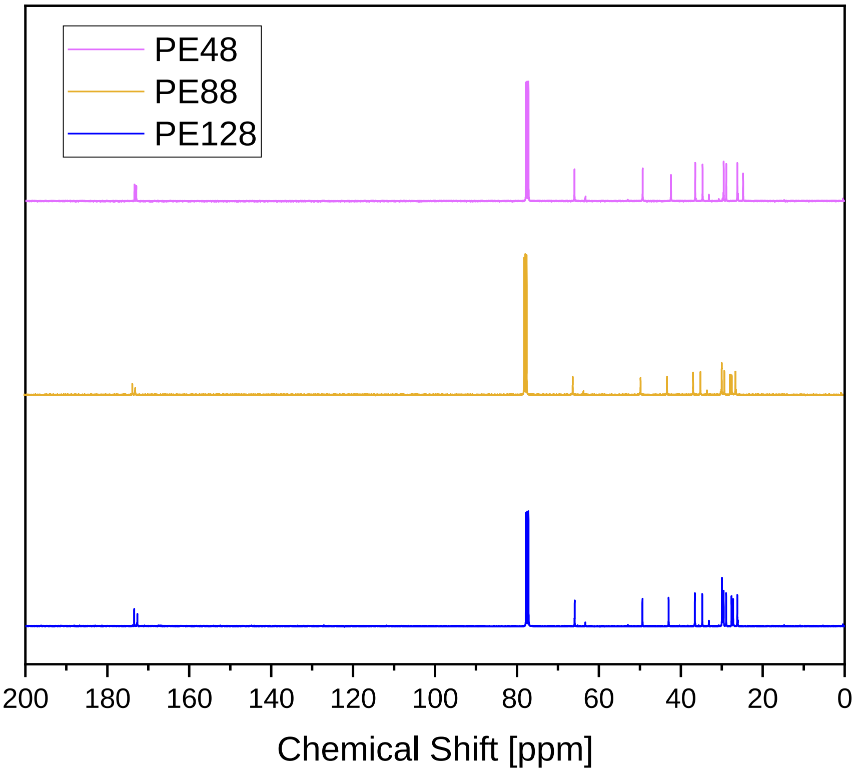

Figure S9. ^{13}C-NMR of PE48, PE88, PE128.

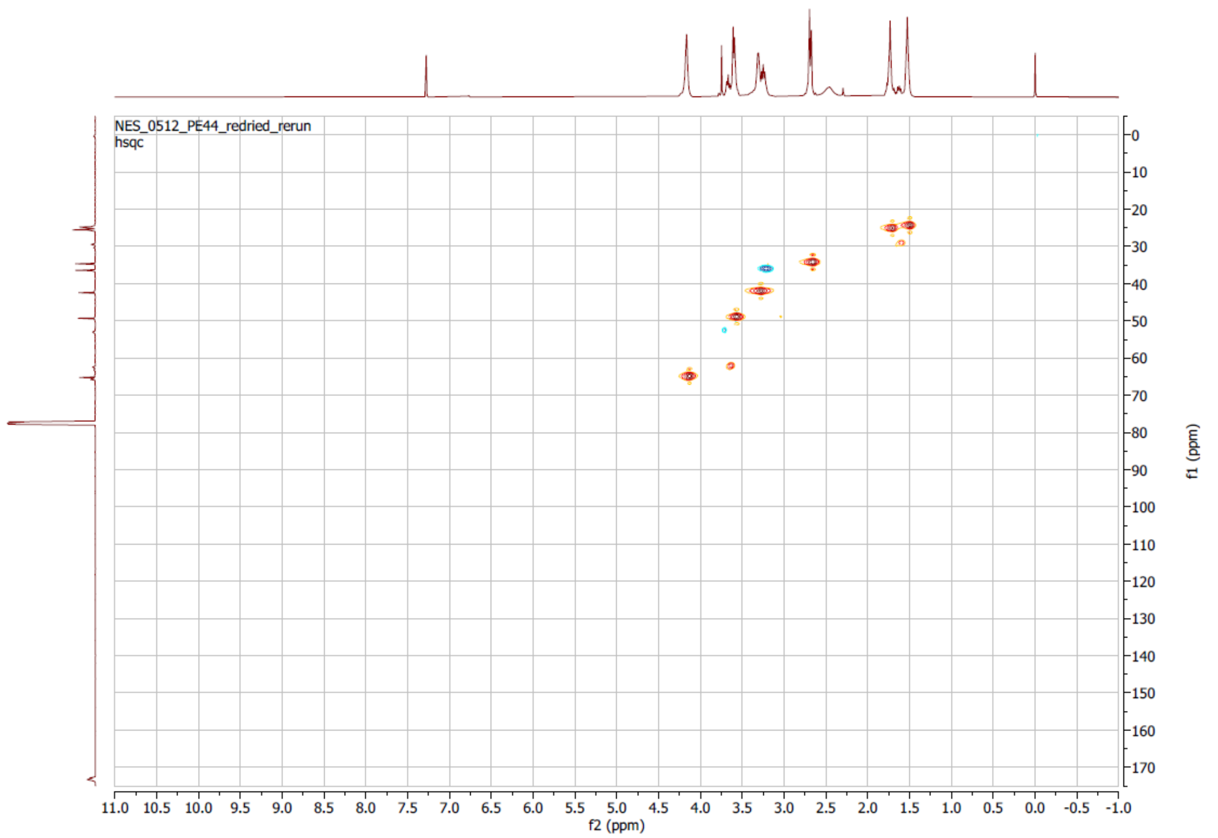

Figure S10. HSQC (^{1}H-^{13}C APT)-NMR of PE44.

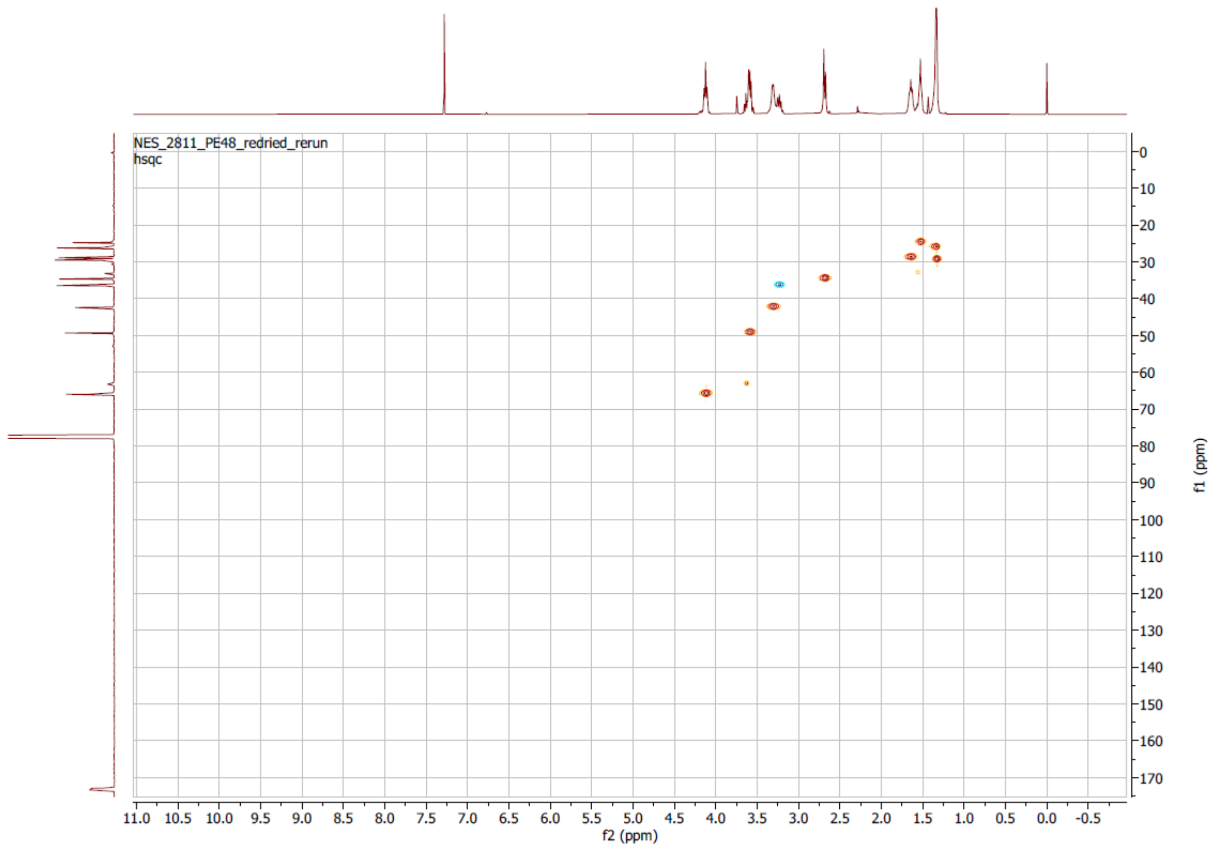

Figure S11. HSQC (^{1}H-^{13}C APT)-NMR of PE48.

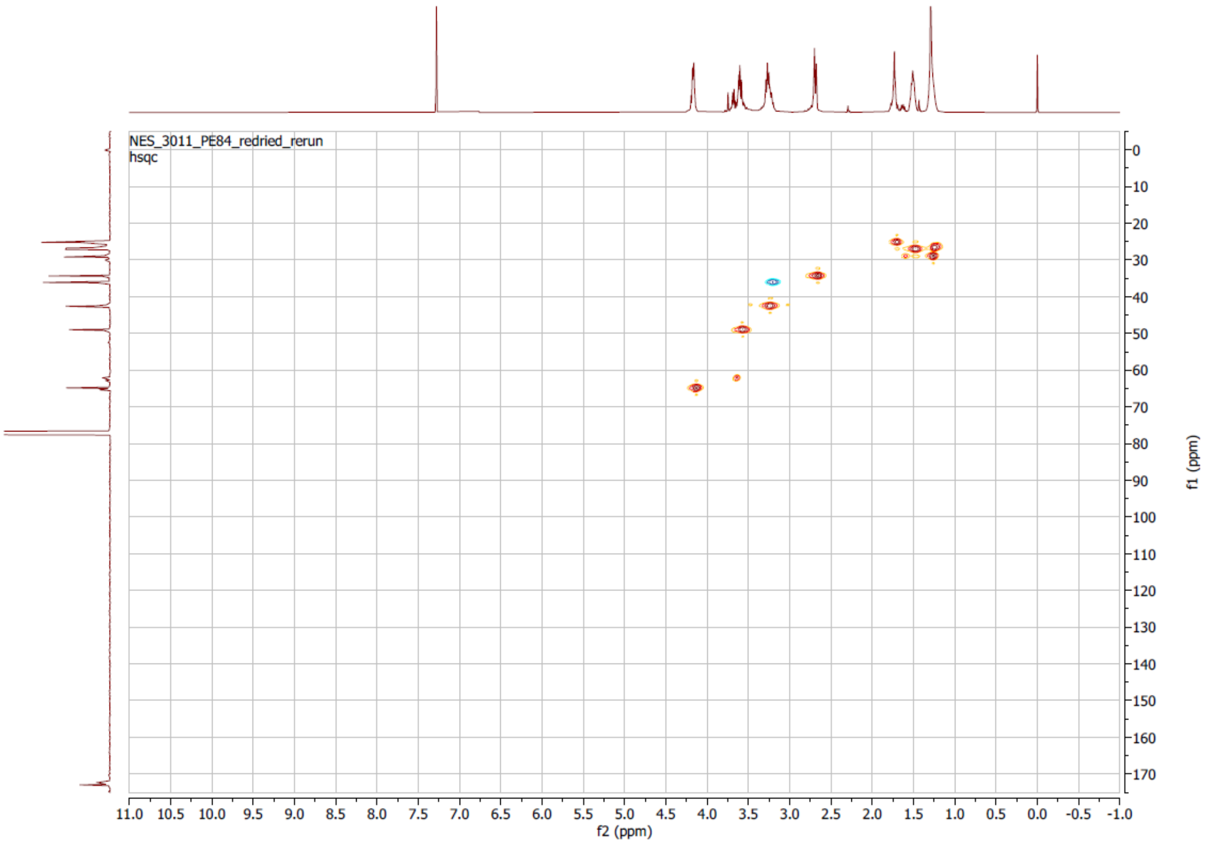

Figure S12. HSQC (^{1}H-^{13}C APT)-NMR of PE84.

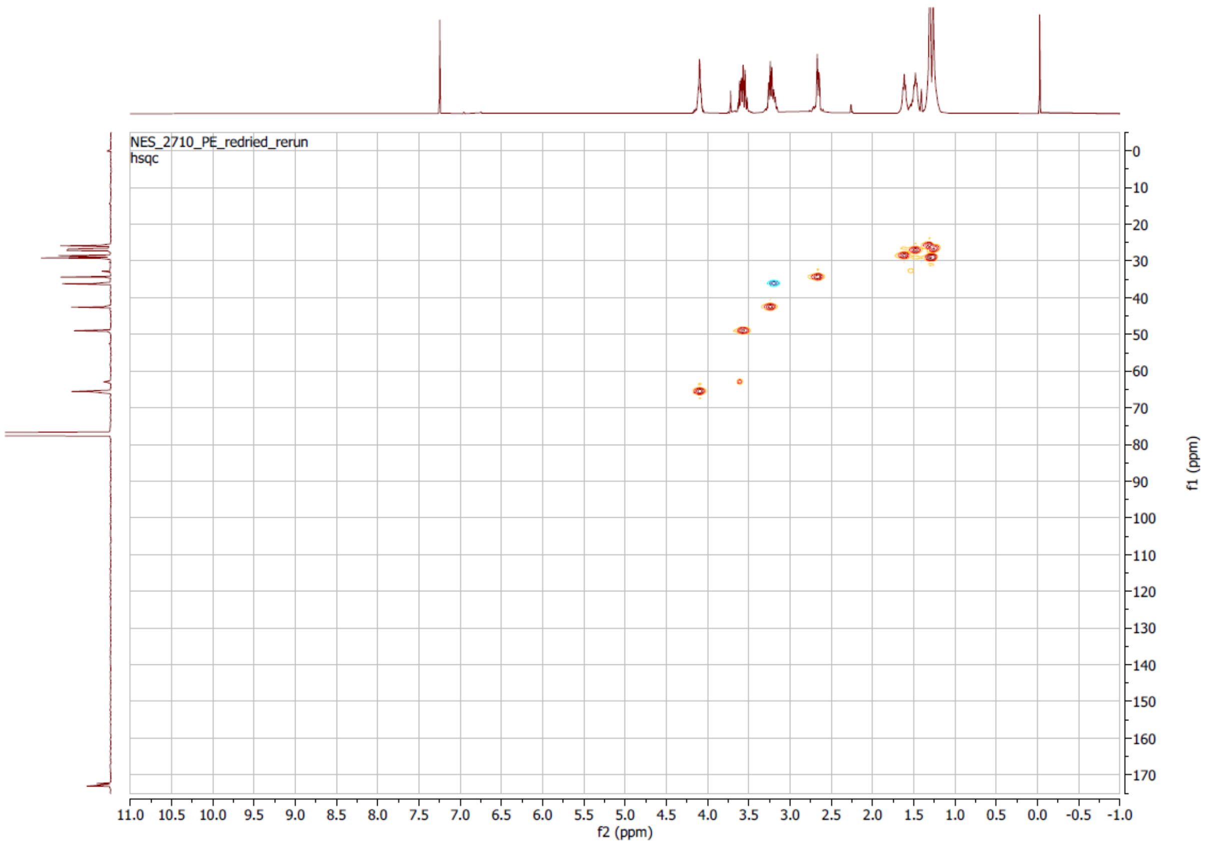

Figure S13. HSQC (^{1}H-^{13}C APT)-NMR of PE88.

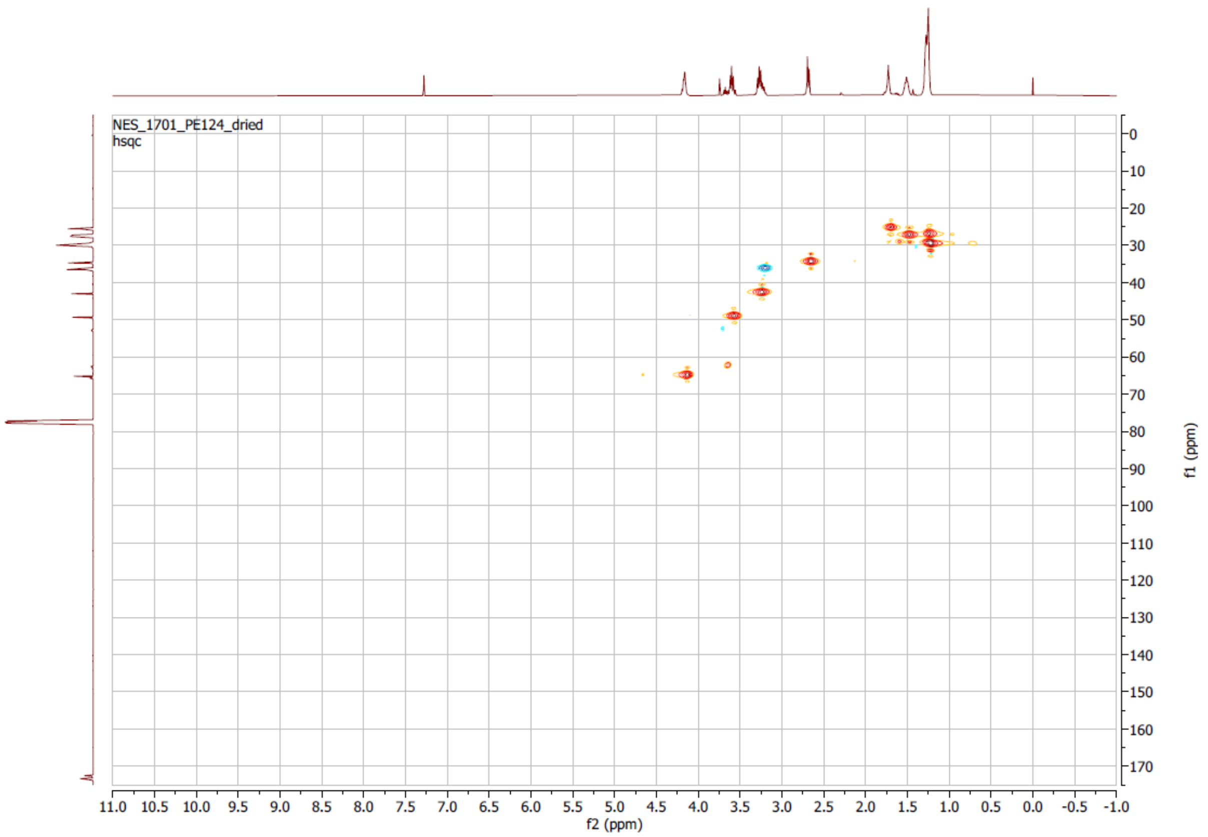

Figure S14. HSQC (^1H-^{13}C APT)-NMR of PE124.

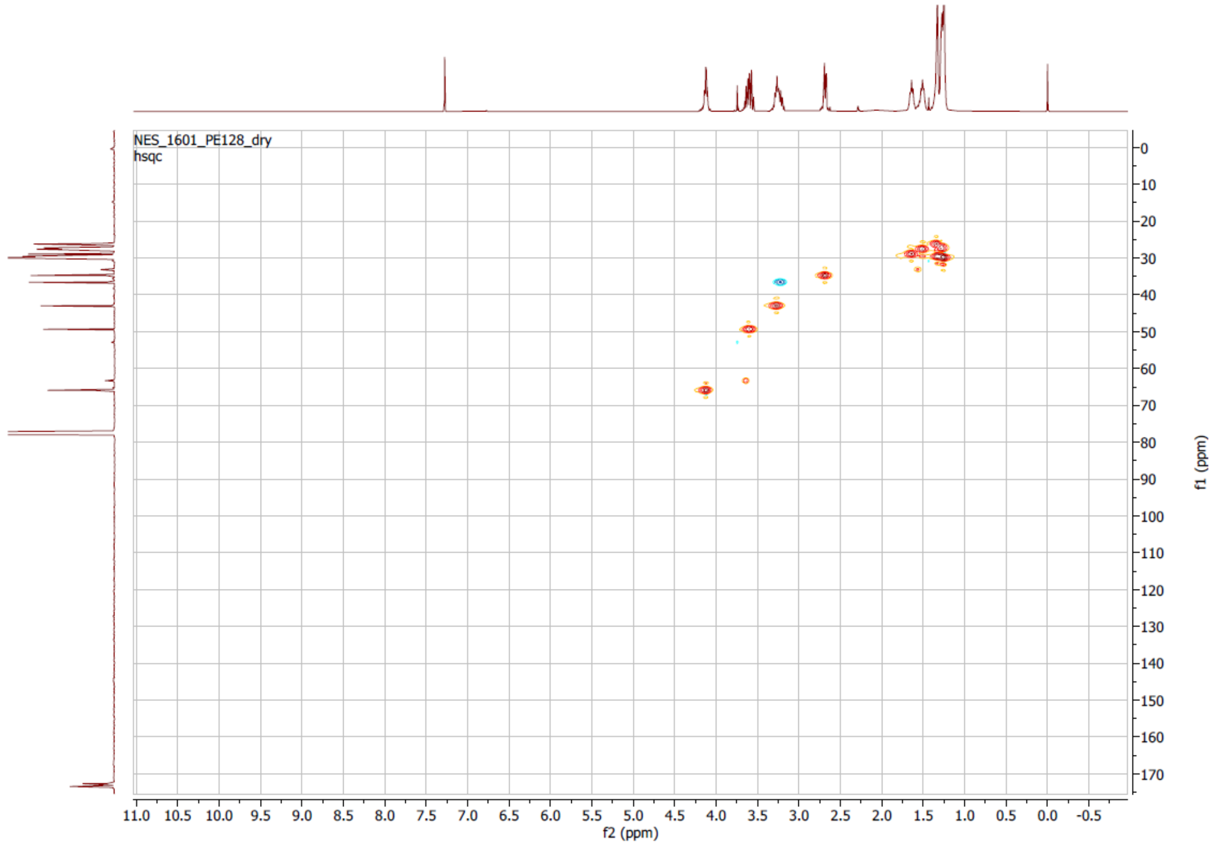

Figure S15. HSQC (^1H-^{13}C APT)-NMR of PE128.

2. GC-MS Analysis

Table S1. Calculated mass-to-charge-Ratio (m/z) of itaconic-based monomers (BPdm, DAMS, and DOMS).

**Monomer**	**Molecular Formular**	**M_w [g/mol]**	**m/z**
C4BPdm	$C_{16}H_{24}N_2O_6$	340.37	340.16
C8BPdm	$C_{20}H_{32}N_2O_6$	396.48	396.23
C12BPdm	$C_{24}H_{36}N_2O_6$	452.58	452.59

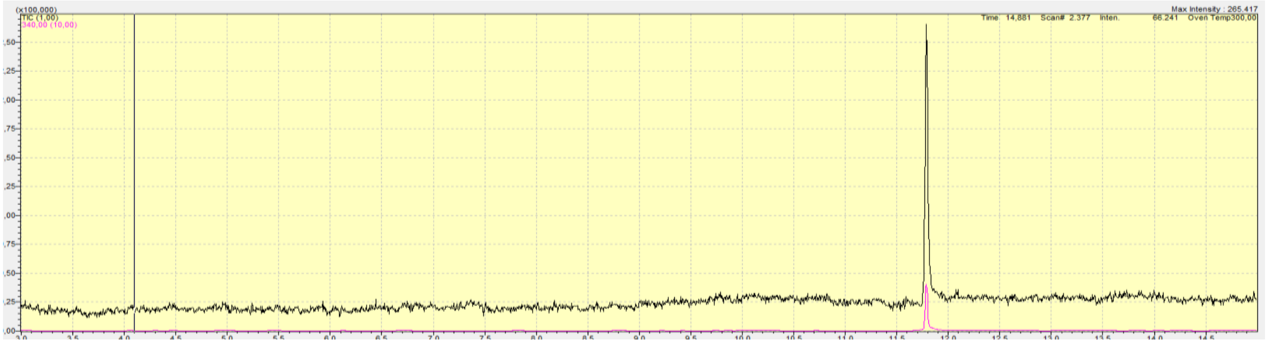

Figure S16. GC-MS chromatogram (black) and extracted molecular ion (pink) at 340 m/z of the synthesized C4BPdm. The single peak which eluted after 11.7 min confirms the high purity already evidenced in the ^{1}H-NMR analysis.

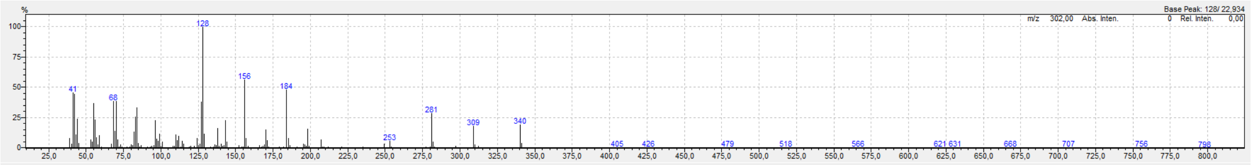

Figure S17. MS of peaks detected at 11.79 min (retention time of compound) during GC-MS of C4BPdm.

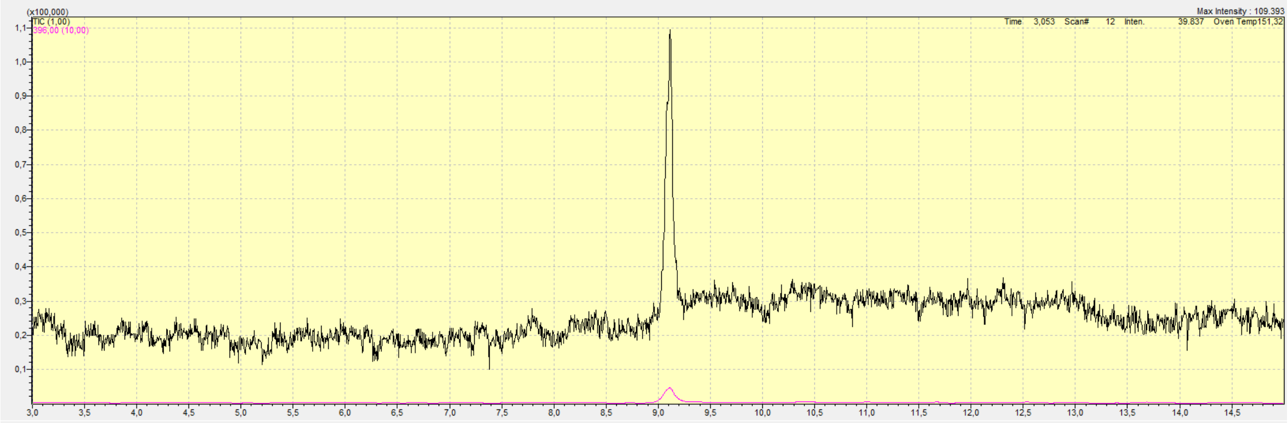

Figure S18. Extracted ion chromatogram of m/z from GC-MS of C8BPdm. The black line shows the experimental data, the pink line the extracted signal corresponding to 396m/z.

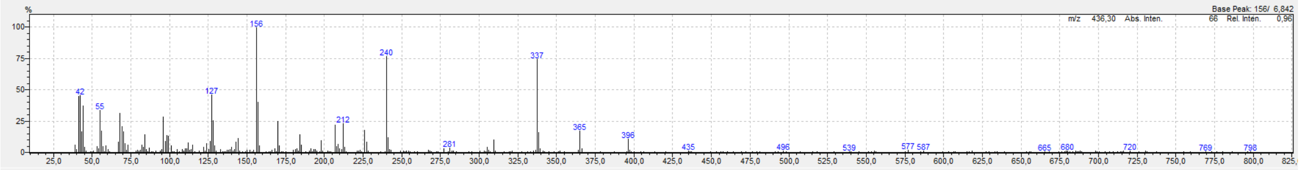

Figure S19. MS of peaks detected at 9.11 min (retention time of compound) during GC-MS of C8BPdm.

3. HPLC-MS Analysis

Table S2. Calibration standard (sodium formate solution 1 mg/mL in 2-propanol/water 1:1) over the range m/z 50–800 was used to autocalibrate the mass with reference calibration masses.

Peak number	Positive Ionisation m/z	Negative Ionisation m/z
Peak 1	90.98	112.99
Peak 2	158.96	180.97
Peak 3	226.95	248.96
Peak 4	362.92	384.93
Peak 5	430.91	452.92
Peak 6	498.9	520.91
Peak 7	566.89	588.9
Peak 8	634.88	656.88
Peak 9	702.86	724.87
Peak 10	770.85	792.86

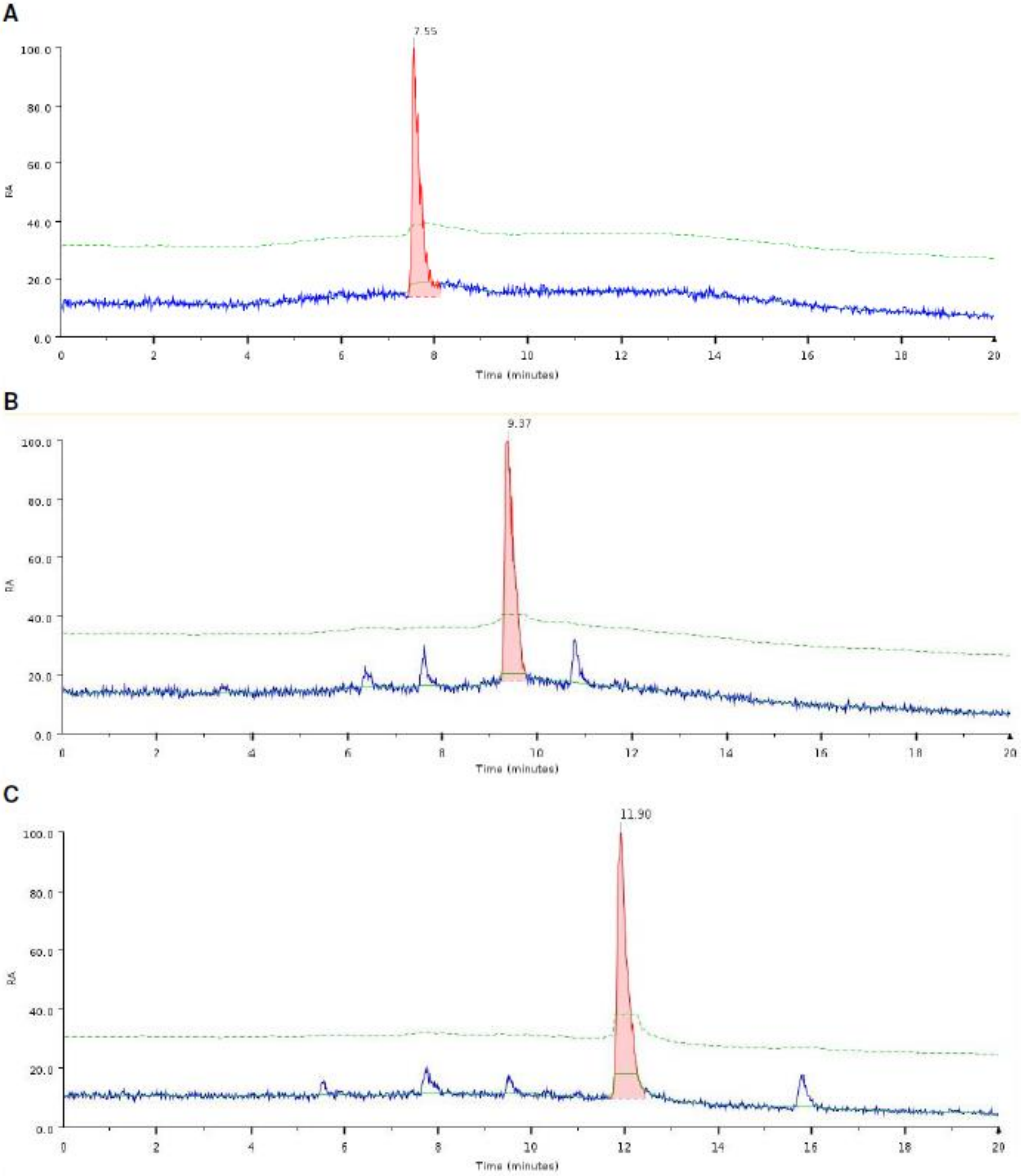

Figure S20. Chromatograms showing the relative absorbance (RA) in relation to time of elution time of BPdm compounds produced in this work: **A**: HPLC chromatogram of C4BPdm; **B**: HPLC chromatogram of C8BPdm; **C**: HPLC chromatogram of C12BPdm.

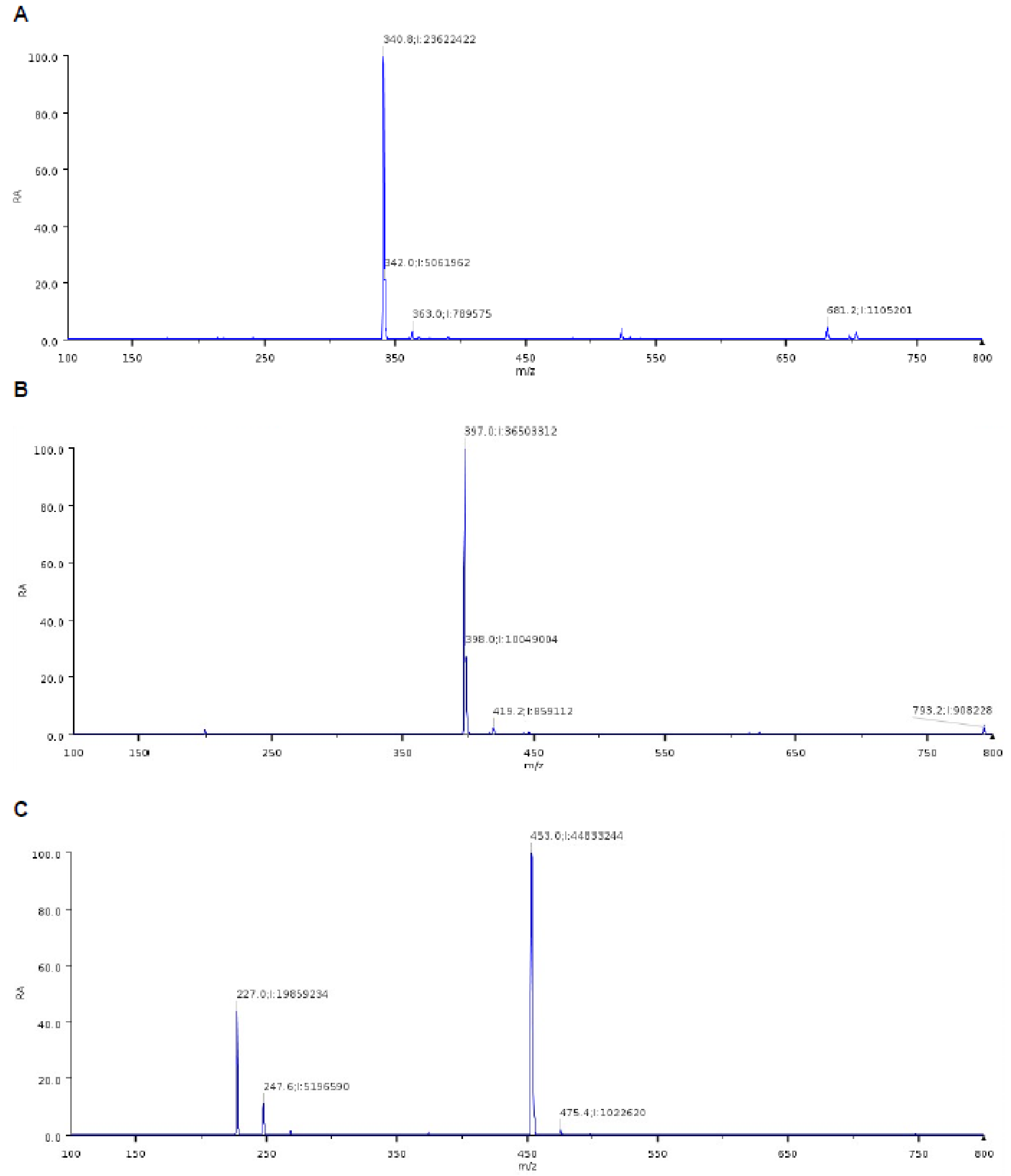

Figure S21. Mass spectra showing the relative absorbance (RA) in relation to mass-to-charge (m/z) of BPdm compounds produced in this work: **A**: HPLC-MS spectrum of C4BPdm showing the C4BPdm compound at 340.8 m/z, C4BPdm + 1 proton at 342.0 m/z, C4BPdm + sodium (22) at 363.0 m/z and C4BPdm as dimer at 681.2 m/z; **B**: HPLC-MS spectrum of C8BPdm with the C8BPdm molecule at 397.0 m/z, and C8BPdm + 1 proton at 398.0 m/z, as well as in complex with sodium at 419.2 m/z, and as dimer at 793.2 m/z; **C**: HPLC-MS spectrum of C12BPdm showing the compound itself at 453.0 m/z, C12BPdm + sodium at 475.4 m/z. The dimer of C12BPdm is not observed as it falls out of the analyzed spectrum (>800 m/z), however, a fragmentation of the compound is indicated by two peaks at 227.0 m/z and 247.6 m/z (the sum of which form ~ 475 m/z corresponding to C12BPdm + sodium).

4. FT-IR Analysis

Analysis of Monomers

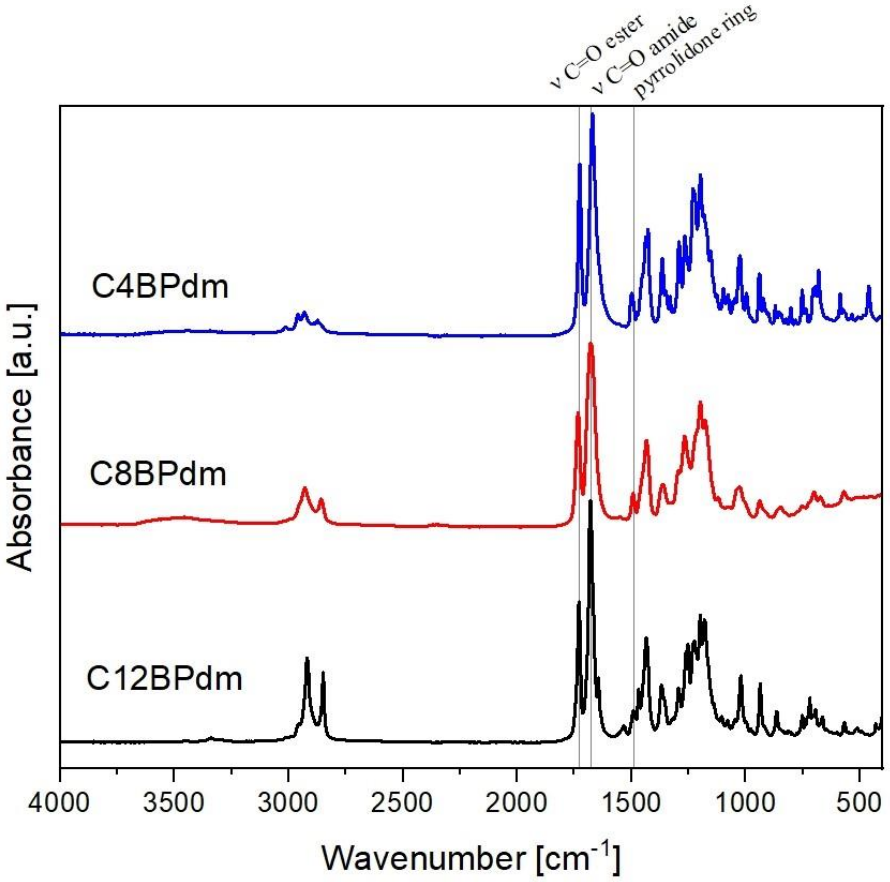

Figure S22. IR spectra for monomers C4BPdm (blue), C8BPdm (red), C12BPdm (black), collected at room temperature.

Analysis of Polymers

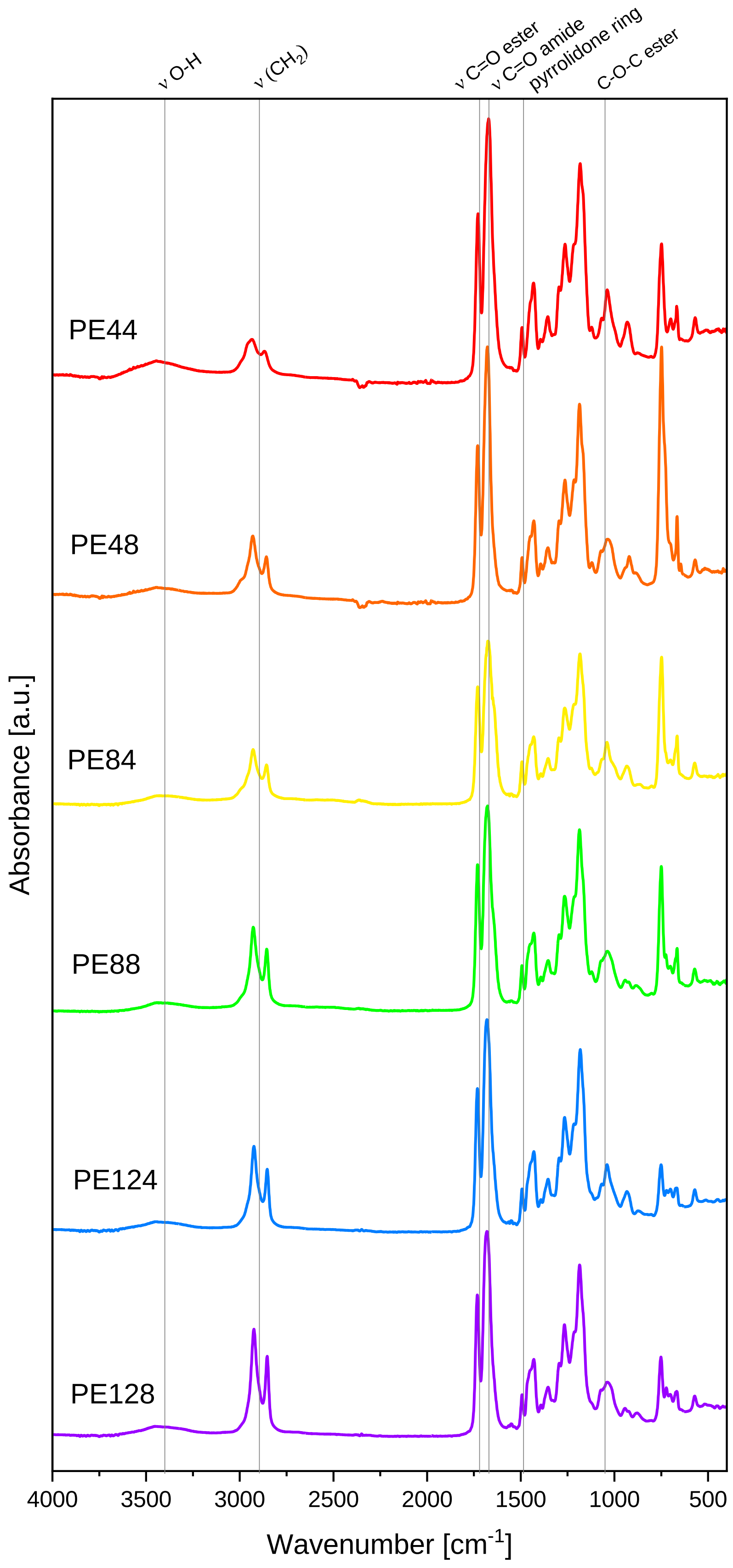

Figure S23. FT-IR spectra for polymers PE44 (light blue), PE48 (green), PE84 (dark blue), PE88 (purple), PE124 (pink), PE128 (orange), collected at room temperature.

5. TGA Analysis

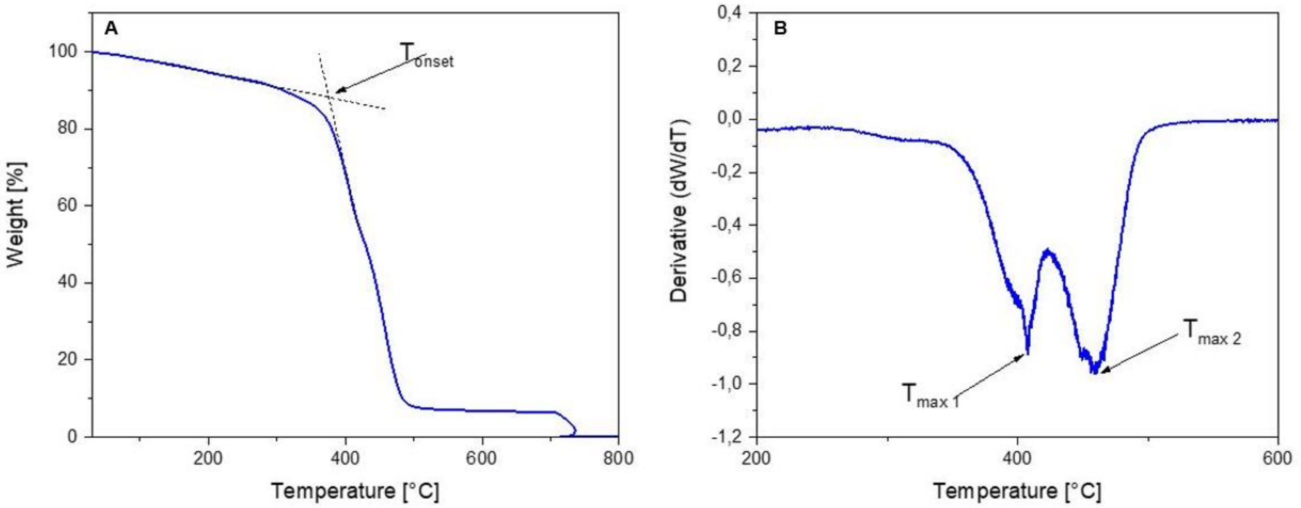

Figure S24. Schematic TGA curve showing the thermal degradation of sample of PE84. **A**: The T_{onset} was derived from the TG. **B**: T_{max1} and T_{max2} were obtained by analyzing the first derivative of TG, the DTG curve. This compound in example shows some initial evaporation followed by a two-step thermal decomposition leaving a char residue which is subsequently removed during oxygen purging phase after 700 °C.

Analysis of Monomers

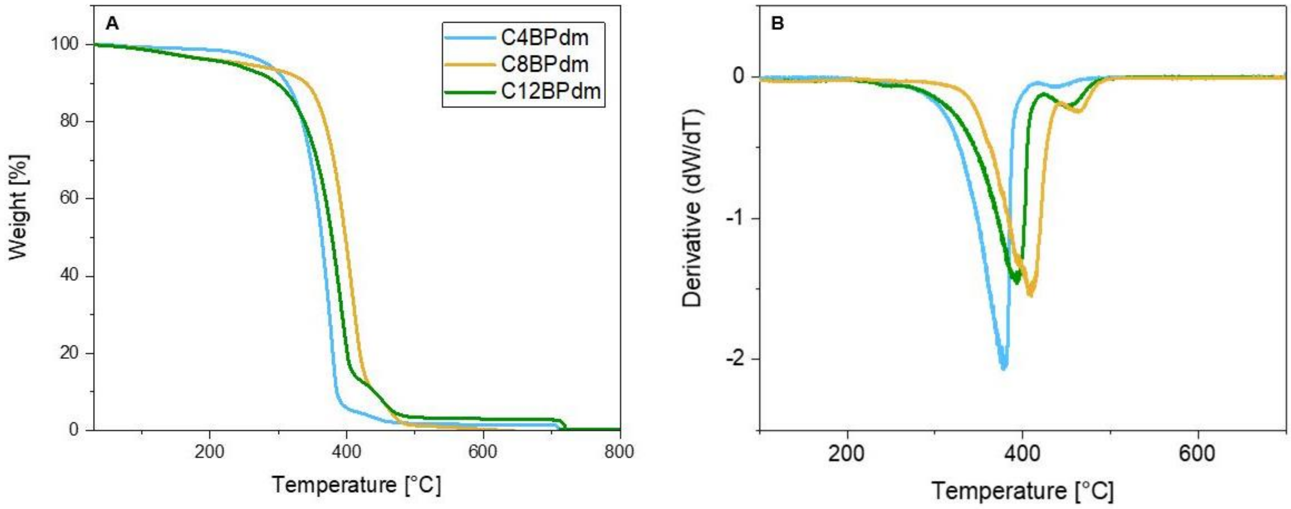

Figure S25. TGA of monomers C4BPdm (light blue), C8BPdm (deep green) and C12BPdm (orange). A: Thermal degradation of BP-dm monomers occurs above 380 °C. (C4BPdm TD_{10}: 385.5°C, TD_{50}: 363.4°C; C8BPdm TD_{10}: 418.5°C, TD_{50}: 381.8°C; C12BPdm TD_{10}: 440.4°C, TD_{50}: 397.6°C). B: The first derivative of the curves (DTG) is shown on the right side.

Analysis of Oligomers

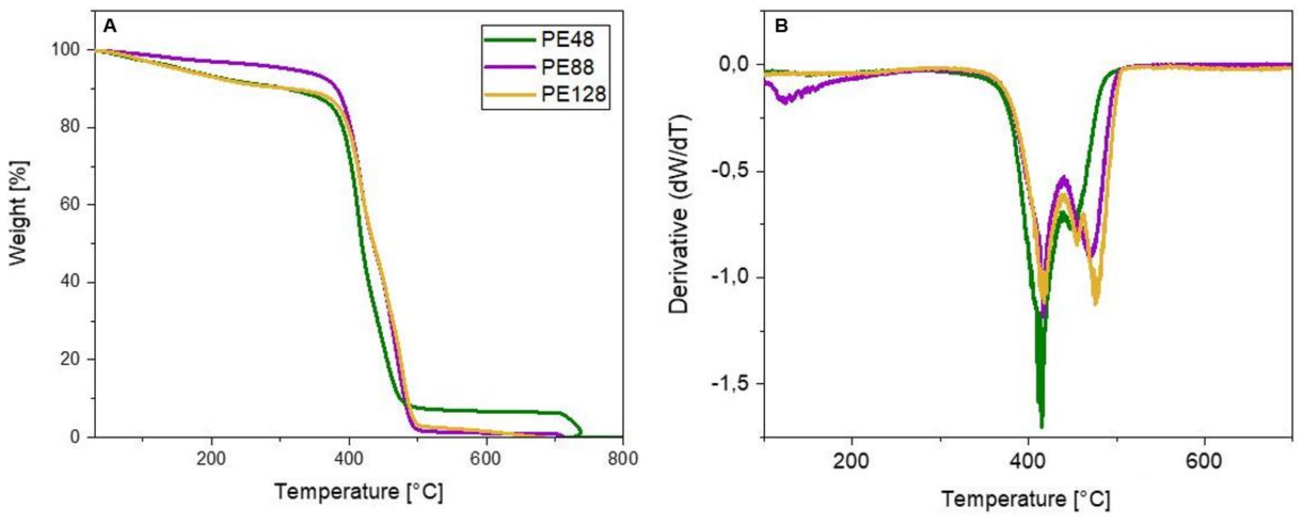

Figure S26. TGA (A) and DTG (B) of polymers PE44 (light blue), PE84 (dark blue), and PE124 (pink).

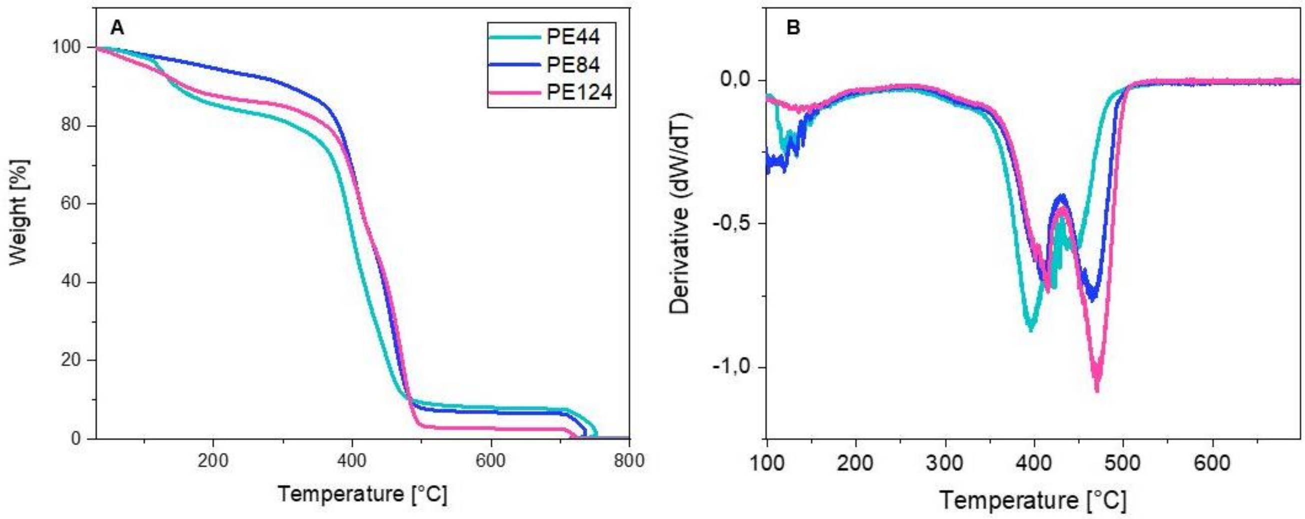

Figure S27. TGA of polymers PE48 (green), PE88 (purple), and PE128 (orange). **A:** TGA of PE*x*8; **B**: DTG of PE*x*8.

Analysis of PLA Formulations

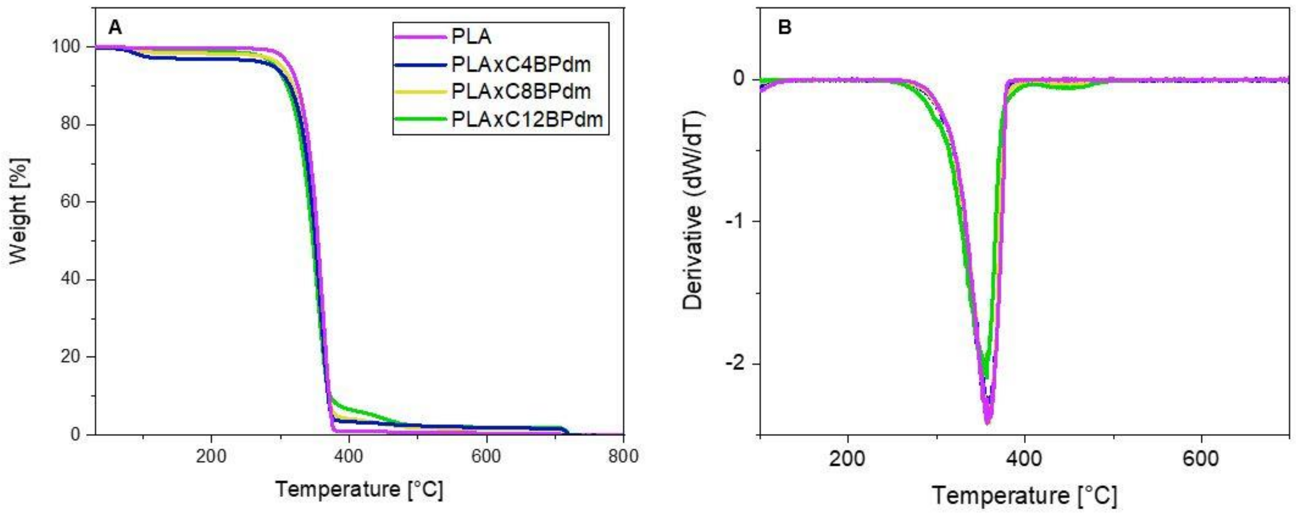

Figure S28. Thermal analysis of polymer formulations: PLA blank formulation (purple), PLAxC4BPdm (blue), PLAxC8BPdm (yellow), PLAxC12BPdm (green). **A:** TGA of formulations; **B**: DTG of formulations.

6. DSC Analysis

Analysis of Monomers

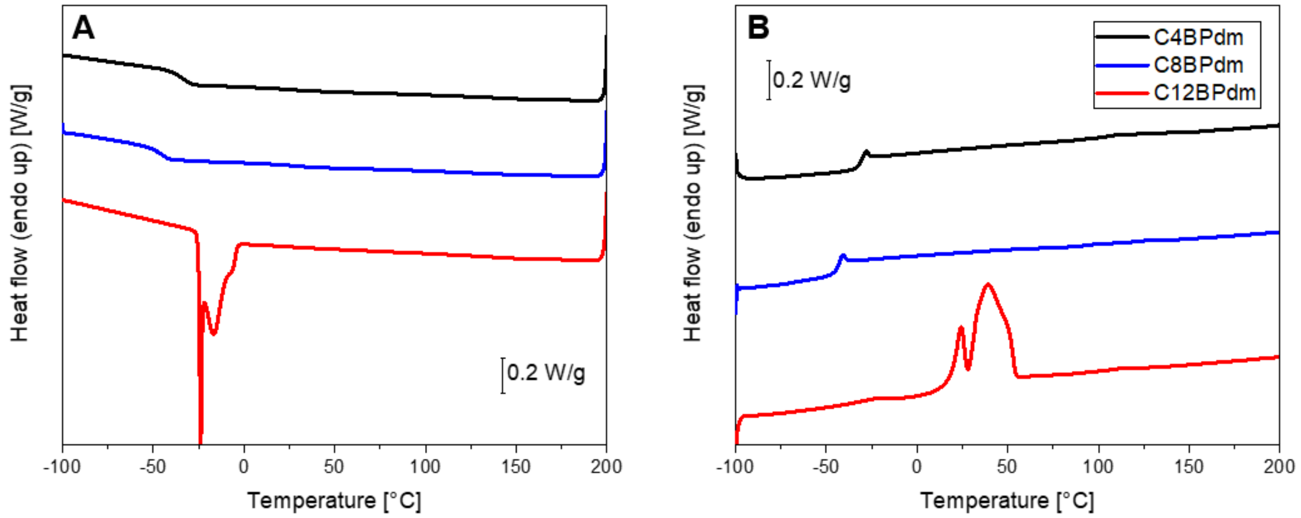

Figure S29. DSC thermograms of BPdm-monomers C4BPdm, C8BPdm, and C12BPdm. The DSC analysis for C4BPdm (black), C8BPdm (blue), and C12BPdm (red) show a significant difference in terms of T_g. **A**: cooling; **B:** second heating run.

Analysis of Polymers

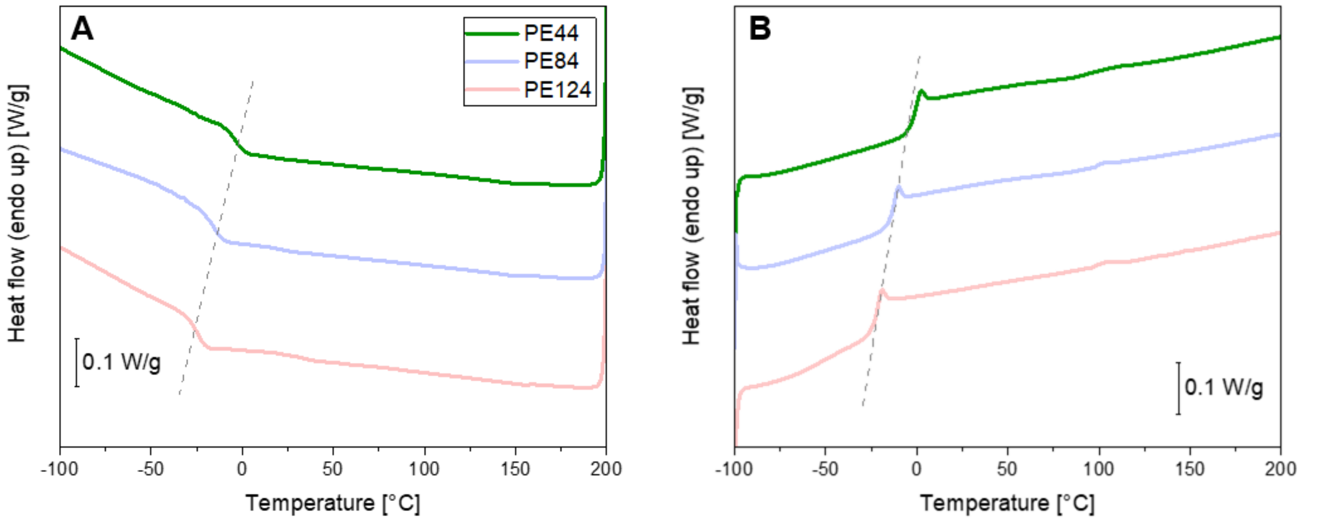

Figure S30. DSC thermograms of BDO-based polyester PE44 (green), PE84 (blue), and PE124 (rosé) display the effect of the bis-pyrrolidone monomer in T_g for the BP-based polymers with the linear aliphatic BDO. **A**: cooling; **B**: second heating run.

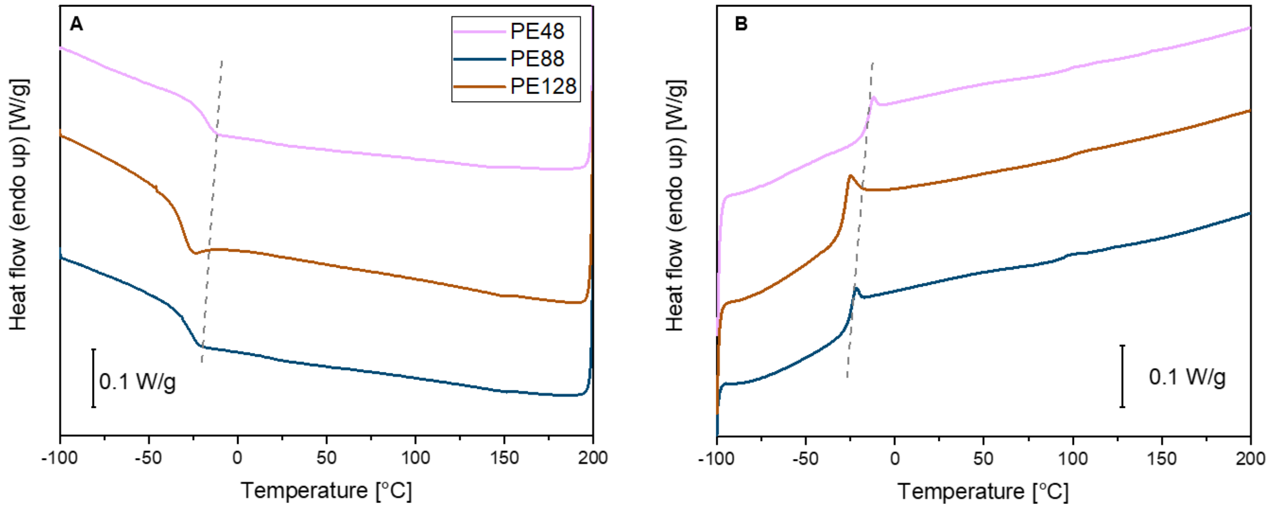

Figure S31. DSC thermograms of ODO-based polyester PE48 (pink), PE88 (blue), and PE128 (brown) display the effect of the bis-pyrrolidone monomer in T_g for the BP-based polymers with the linear aliphatic ODO. **A**: cooling; **B**: second heating run.

Table S3. GPC, DSC and TGA analysis of the functional polyesters synthesized in this work. The bis-pyrrolidone structures were reacted with two different diols (BDO, ODO) using a solventless-enzymatic polycondensation approach.

**Sample**	***M₀*** [g mol⁻¹]	M_n [a] [g mol⁻¹]	M_w [a] [g mol⁻¹]	*Đ* [a]	*DP* [b]	T_g [c] [°C]	T_{onset} [d] [°C]	$T_{max\,1}$ [d] [°C]	$T_{max\,2}$ [d] [°C]	*Conv.* [e] [%]
PE44	366.40	800	1000	1.21	2	-2	365	396	448	72
PE48	422.51	2100	3800	1.79	5	-16	391	415	458	85
PE84	422.51	1400	2600	1.94	3	-14	387	413	467	84
PE88	478.62	1300	3000	2.23	3	-25	393	417	472	82
PE124	478.61	2400	4900	2.04	5	-23	384	416	471	92
PE128	534.72	3300	6500	1.98	6	-29	397	416	477	98

[a] calculated from GPC analysis; [b] calculated *via* M_n/M_0; [c] calculated from DSC analysis; [d] calculated from TGA analysis. [e] calculated from ^{1}H-NMR using **Eq. (2)**.

Analysis of PLA Formulations

The solvent casted films were initially analyzed after three days of drying in the vacuum oven. However, it was quickly understood that the PLAxC*x*BPdm films retained the solvent, which has a known plasticizing effect on PLA, for more than three days. Mechanical tests were run after the film reached a constant weight equal to the weighted in compounds, which was after 6 days.

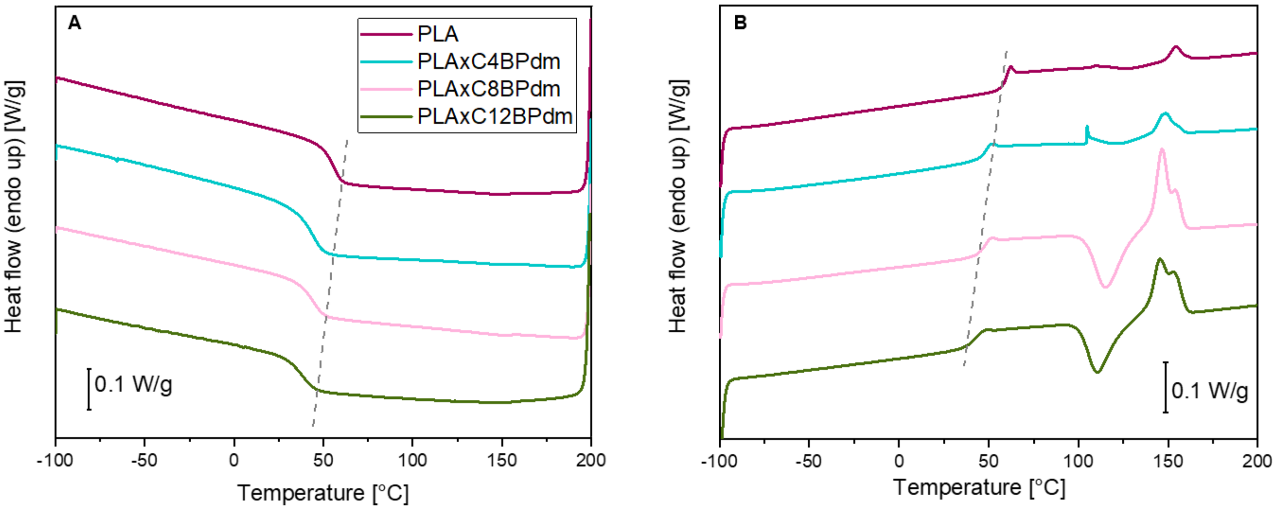

Figure S32. DSC thermograms of PLA formulations with BPdm-monomers C4BPdm, C8BPdm, and C12BPdm. The DSC analysis for PLAxC4BPdm (turquoise), PLAxC8BPdm (pink), and PLAxC12BPdm (green) show a significant difference in terms of T_g of the neat PLA (violet). This proves that the CxBPdm monomers have a plasticizing effect on PLA. **A**: cooling; **B**: second heating run.

7. GPC Analysis

Table S4. GPC analysis of the functional polyesters synthesized in this work. Three different bis-pyrrolidone structures were reacted with two different diols (BDO, ODO) in a chemo-enzymatic polycondensation reaction.

**Sample**	**M_0** *[g mol^{-1}]*	**M_n[a]** *[g mol^{-1}]*	**μ(M_n)** *[g mol^{-1}]*	**M_w[a]** *[g mol^{-1}]*	**μ(M_w)** *[g mol^{-1}]*	**Đ[a]**	**μ(Đ)**	**DP[b]**	**μ(DP)**
PE44	366.40	846	810	1034	976	2.31	2.21	1.22	1.21
		774		918		2.11		1.19	
PE48	422.51	2176	2128	3310	3784.5	4.92	5.04	1.52	1.79
		2079		4259		5.15		2.05	
PE84	422.51	1443	1357	2574	2611.5	3.42	3.22	1.78	1.94
		1270		2649		3.01		2.09	
PE88	478.62	1231	1340.5	2971	2967.5	2.57	2.8	2.41	2.23
		1450		2964		3.03		2.04	
PE124	478.61	2300	2399	4924	4881	4.81	5.02	2.14	2.04
		2498		4838		5.22		1.94	
PE128	534.72	3641	3324	7121	6545.5	5.62	6.22	1.96	1.98
		3006		5970		6.81		1.99	

[a] calculated from GPC analysis. [b] calculated via M_n/M_0; Note: The weight of the repetitive unit of the polymer (M_0) was calculated based on the theoretical structure of the respective repetitive unit. The mean (μ) of the number-average molecular weight (M_n) and of the weight-average molecular weight (M_w) were calculated based on M_n and M_w from the GPC analysis of two samples of the same reaction set up performed in duplicate.

8. Stress Strain Analysis

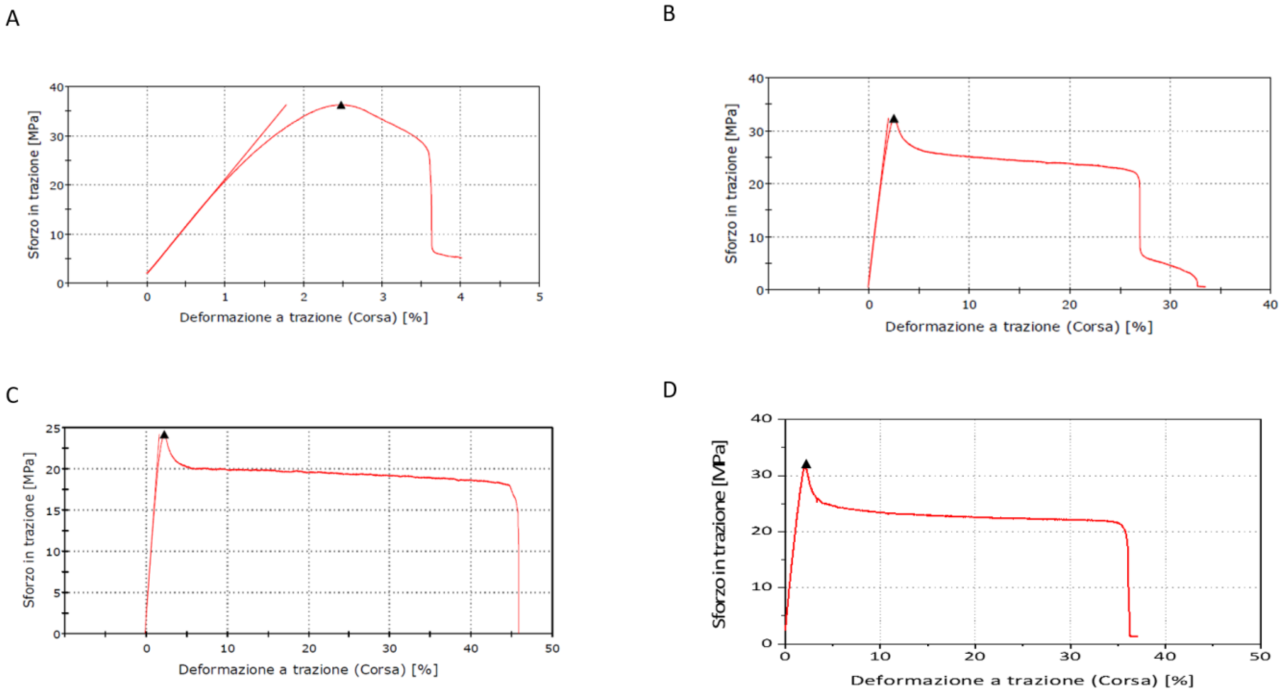

Figure S33. Representative stress-strain curves of the tested PLA formulations. **A:** the neat PLA; **B:** PLAxC4BPdm; **C:** PLAxC8BPdm; and **D:** PLAxC12BPdm. The yield strength of each sample is marked by a small black triangle.
